# PEC-CDC: A prediction error-based calibration framework for robust unsupervised deep clustering

**DOI:** 10.1371/journal.pone.0351959

**Published:** 2026-06-25

**Authors:** Ziyang Li

**Affiliations:** College of Art, Northeast Agricultural University, Heilongjiang, Harbin, China; Donghua University, CHINA

## Abstract

While deep clustering has made significant strides, the inherent overconfidence of Softmax-based calibration remains a critical bottleneck. Even dedicated frameworks like Calibrated Deep Clustering (CDC) fail to fully eliminate this issue, as they continue to rely on probability-based metrics. These relative metrics are constrained by inter-class normalization, which often forces high confidence scores onto ambiguous samples where discriminative features are underdeveloped. To address this limitation, we propose PEC-CDC, a novel framework that reconfigures the CDC architecture by replacing its probability-based calibration head with a regression-based Prediction Error-based Classification (PEC) head. Our core motivation is that prediction error serves as an absolute measure of distributional support independent of other classes, making it inherently resistant to overconfidence. Specifically, we utilize a frozen Teacher-Student module to shift the model’s evaluation from relative confidence probability to absolute sample–cluster distribution consistency. Qualitative analysis via t-SNE and error distribution visualizations confirms that our framework establishes superior semantic boundaries and compact cluster manifolds. Extensive experiments demonstrate that PEC-CDC achieves state-of-the-art performance; notably, on the challenging CIFAR-100 dataset, it attains an Adjusted Rand Index (ARI) of 45.61%, effectively quadrupling the performance of leading baselines such as CueCo.

## Introduction

In the era of data-driven intelligence, we are witnessing an unprecedented explosion in data volume and complexity across domains such as satellite imagery, biomedical imaging, and multimedia archives. This exponential growth contrasts sharply with the high cost, scarcity, and inherent inefficiency of manual data annotation [1]. Consequently, unsupervised learning has emerged as a pivotal and increasingly urgent research direction within machine learning, aiming to extract meaningful patterns from data without relying on costly labels [2,3]. As a core branch of this paradigm, unsupervised clustering specifically seeks to uncover inherent group structures and latent patterns directly from raw, unlabeled data. This capability underpins a wide spectrum of critical applications, ranging from organizing large-scale visual datasets and enabling content-based image retrieval in computer vision to topic discovery and document organization in natural language processing [4]. The field has evolved significantly from classic distance-based algorithms like K-means to sophisticated modern approaches, including deep embedded clustering [5] that learns feature representations, and contrastive learning-based methods like SCAN [6,7] that leverage instance discrimination. Despite these advancements, ensuring reliable, robust, and semantically coherent clustering results in the complete absence of supervisory signals remains a critical challenge, especially when the training process is affected by noisy samples, uncertain early-stage assignments, and ambiguous class boundaries.

The self-training framework, centered on pseudo-label generation and iterative model optimization, has become a mainstream approach to enhancing unsupervised clustering performance [8,9]. Literature clearly indicates that leveraging unlabeled data by iteratively assigning pseudo-labels constitutes the core principle of self-training [10]. This paradigm has been widely applied and optimized across numerous specific studies, such as constructing iteratively refined pseudo-label generation frameworks for novel intent discovery tasks, and improving mainstream self-training approaches in unsupervised domain adaptation through adaptive pseudo-labeling strategies [11,12]. However, effectiveness is fundamentally constrained by the quality of pseudo-labels; noisy labels can misguide the learning process, trapping the model in local optima and leading to performance degradation [13]. In this context, robustness refers to the ability of the model to avoid selecting unreliable pseudo-labels under noisy or uncertain training conditions, including noisy samples, early-stage misassignments, and samples near ambiguous class boundaries. Confidence calibration is therefore key to pseudo-label assessment. Yet most existing methods rely on softmax-derived probabilities, which suffer from inherent overconfidence: models frequently assign high confidence to incorrect predictions, especially when discriminative structures are underdeveloped or class boundaries are ambiguous [14]. Once such overconfident but incorrect pseudo-labels are selected, their errors can be reinforced in later iterations, reducing optimization stability and increasing the risk of poor local optima. This miscalibration poses a significant barrier to obtaining reliable pseudo-labels in fully unsupervised settings.

To address this issue, the Calibrated Deep Clustering (CDC) framework introduced a dedicated calibration head to refine softmax confidence, achieving linear complexity through graph filtering and adaptive anchors [15]. However, CDC remains dependent on softmax probabilities and fails to eliminate overconfidence when class boundaries are unclear [16,17]. Indeed, existing pseudo-label refinement methods, whether image clustering approaches such as SPICE [18] or strategies that adopt iterative cooperation or optimal transport like RoNID [11], have not yet managed to move beyond the framework of probability metrics. This limitation suggests that, to improve robustness during iterative self-training, confidence estimation should not only calibrate probability scores but also reduce the selection of unreliable pseudo-labels. During learning, noisy samples, early-stage misassignments, and samples near ambiguous class boundaries may still be assigned high-confidence pseudo-labels. Existing probability-based refinement methods, including calibrated deep clustering methods, derive confidence from relative class probabilities. Thus, even after calibration, a sample may be regarded as reliable simply because one class score is higher than the others, although its cluster membership remains uncertain. Such unreliable pseudo-labels may be repeatedly reused in subsequent optimization, amplifying errors and reducing training stability. A more robust calibration mechanism should therefore evaluate whether a sample conforms to the distributional structure of a specific cluster, rather than only whether it obtains a relatively high probability.

We identify the Prediction Error-based Classification (PEC) method as a specific solution to this issue. The core premise is that if a model is sufficiently trained on the data distribution of a certain class, its prediction error near that distribution should be significantly smaller than its error outside the distribution. The prediction error adopted by PEC is an absolute quantity, independent of inter-class relative normalization. Compared with probability-based confidence, prediction error evaluates whether a sample is well supported by a cluster-specific distribution rather than whether it merely obtains the largest relative probability. Therefore, samples affected by noise or located near ambiguous boundaries tend to produce larger errors and are less likely to be selected as reliable pseudo-labels. As a result, it possesses inherent advantages: it is less prone to overconfidence, more closely aligned with uncertainty measurement, and highly compatible with clustering scenarios [19].

Prediction error is increasingly recognized as a reliable indicator of uncertainty across various domains. Within class-incremental learning, frameworks comprising a frozen teacher coupled with a student network utilize prediction error as a scoring criterion, a strategy that has proven highly effective [20]. Recent deep clustering research has also verified that prediction error correlates with cluster distribution consistency, supported by its theoretical connection to Gaussian process posterior variance [21]. Inspired by the successful application of prediction error in incremental learning [15,20], this paper proposes the PEC-CDC method. Building upon the complete CDC self-training framework, we introduce a key modification: the CluHead is retained as the core clustering prediction head, while the original Calibration Head is replaced with a newly designed PEC module. The output of the PEC module is no longer a normalized probability distribution but explicitly represents the prediction error, indicating the matching degree between a sample and each cluster distribution. Through this design, the core evaluation mechanism of the model shifts from relying on relative confidence probability derived from softmax outputs to quantifying sample–cluster distribution consistency based on the absolute metric of prediction error. This shift makes pseudo-label selection less sensitive to noisy samples, early-stage misassignments, and ambiguous decision boundaries, thereby improving the robustness of self-training. This change mitigates the unreliability of confidence assessment based on softmax at the source mechanism level.

The remainder of this paper is organized as follows: Chapter 2 reviews relevant research; Chapter 3 details the framework and design of PEC-CDC; Chapter 4 presents experimental validations; Chapter 5 discusses advantages, limitations, and future directions; and Chapter 6 concludes the study.

## Related works

This section reviews four key research areas relevant to the proposed PEC method: unsupervised deep clustering, pseudo-label quality optimization, confidence calibration in clustering, and prediction error-based uncertainty estimation. Existing gaps in current research are also highlighted to contextualize our contributions.

## Unsupervised deep clustering

Clustering research has long been developed through conventional paradigms, such as partition-based clustering, hierarchical clustering, density-based clustering, and graph-based clustering. Representative studies have further improved these paradigms from different perspectives [22–24]. Building on these foundations, the development of unsupervised deep clustering has shifted toward integrating self-supervised learning with advanced optimization strategies [25–27], with pseudo-labeling methods becoming the dominant paradigm [28]. Contemporary approaches typically extract features through self-supervised learning frameworks such as MoCo-v2 [29], attach a clustering head to generate predictions, and use the model outputs as pseudo-labels to guide unsupervised training [15]. Contrastive learning has emerged as a driving force in this field; representative methods such as SimCLR [30], MoCo-v2 [29], and MoCo-v3 [31] enhance feature discriminability by scattering representations across the latent space to prevent dimensional collapse. Building upon these foundations, DINO [32] introduces a teacher-student self-distillation framework where a student network learns to predict the output of a momentum teacher network. This mechanism effectively captures high-level semantic features and utilizes the teacher-student dynamic to generate high-quality positive pairs for training. Furthermore, approaches such as BYOL [33] and SimSiam [34] demonstrate that robust representations can be learned by eliminating negative samples entirely. Similarly, redundancy reduction methods such as Barlow Twins (BT) [35] and VICReg [36] avoid feature collapse by minimizing the cross-correlation between feature dimensions to learn non-redundant and decorrelated representations. Researchers have also analyzed deep clustering methods from the perspective of prior knowledge integration, categorizing approaches into types such as augmentation invariance and neighborhood consistency [4]. Recent innovations like the PLCSR method address the limitations of static thresholding by adopting a curriculum selection strategy with dynamic thresholds, progressively processing nodes through self-reflection to obtain more reliable pseudo-labels [37]. Despite these advances, challenges such as auxiliary task bias and label noise persist, and the overconfidence issue in early training stages remains unresolved.

### Confidence calibration in clustering

Confidence calibration aims to align model-predicted probabilities with actual accuracy, ensuring that high-confidence predictions correspond to high reliability [38]. In unsupervised clustering scenarios, the absence of true labels makes calibration significantly more challenging than in supervised learning [39]. CDC, a representative calibrated deep clustering method, introduces a dedicated CalHead to adjust the overconfident outputs of the CluHead, generating confidence scores that match the learning status of the model [15,40,41]. This framework optimizes pseudo-label selection by leveraging the calibrated confidence distribution, reducing the impact of overconfident predictions on training. However, the CalHead in CDC still relies on softmax probabilities, and the relative normalization property of softmax limits its calibration effectiveness in early training stages [15]. Other works have explored alternative calibration strategies. For example, Guo et al. systematically reviewed confidence calibration methods for unsupervised learning, noting that reliable confidence metrics should be independent of inter-class relative comparisons and proposing that absolute metrics may be more suitable for unsupervised scenarios [38]. Nevertheless, challenges regarding sensitivity to noise, stability across diverse data distributions, and the alignment between error scale and true confidence remain to be addressed and verified in practical applications.

### Advantages of PEC-CDC over representative prior methods

To highlight the advantages of PEC-CDC over representative prior methods, Table 1 provides a summarized comparison in terms of calibration signal, reliance on softmax confidence, robustness to noisy pseudo-labels, and computational overhead. The comparison covers representative contrastive and non-contrastive self-supervised learning (SSL) methods, clustering-oriented deep clustering methods, calibrated deep clustering methods, and our proposed PEC-CDC framework.

**Table 1:** Summarized comparison between PEC-CDC and representative prior methods.

Method	Calibration signal	Softmax reliance	Robustness to noisy pseudo-labels	Overhead
Contrastive SSL methods, e.g., SimCLR, MoCo-v2, MoCo-v3 [29–31]	Contrastive similarity	Low	Indirect	Moderate to high
Non-contrastive SSL methods, e.g., BYOL, SimSiam, VICReg [33,34,36]	Prediction consistency or feature regularization	Low	Not explicit	Low to moderate
Clustering-oriented deep clustering methods, e.g., SwAV, SCAN, SPICE, CueCo [6,7,9,18]	Prototype assignment, neighborhood consistency, or prediction probability	Moderate to high	Moderate	Moderate
Calibrated deep clustering methods [15,41]	Calibrated probability	High	Improved but remains probability dependent	Moderate
PEC-CDC (Ours)	Prediction error	Low	Strong	Moderate

“Indirect” indicates that robustness is obtained mainly through representation learning rather than explicit pseudo-label calibration. “Not explicit” indicates that the method is not specifically designed to address noisy pseudo-label selection.

As summarized in Table 1, the advantages of PEC-CDC mainly arise from its prediction error-based calibration mechanism. Contrastive and non-contrastive SSL methods primarily improve representation quality, but they do not explicitly calibrate pseudo-label reliability; therefore, their robustness to noisy pseudo-labels is only indirect. Clustering-oriented deep clustering methods rely on prototype assignments, neighborhood consistency, or prediction probabilities. When early cluster assignments are unreliable, incorrect pseudo-labels may be repeatedly reinforced during self-training. Calibrated deep clustering methods further improve pseudo-label selection through calibrated probabilities, but they still depend on softmax-derived confidence, which may assign high confidence to ambiguous samples. In contrast, PEC-CDC replaces probability-based calibration with prediction error-based calibration. Since prediction error measures the distributional consistency between a sample and cluster-specific regression models, ambiguous samples with weak distributional support tend to receive lower confidence and are less likely to be selected. Therefore, PEC-CDC improves robustness to noisy pseudo-labels while maintaining moderate computational overhead, as it does not require large-scale pairwise comparisons or global graph construction.

### Preliminaries

In this section, we review the foundational techniques utilized in this study, specifically the Prediction Error-based Classification (PEC) method [20] and the Calibrated Deep Clustering (CDC) framework [15]. For clarity, the primary notations and symbols used throughout this paper are summarized in Table 2.

**Table 2:** Summary of Major Notations.

Symbol	Description
***Data and Preprocessing***	
${𝒟}_{u}$	The training set of unlabeled samples, defined as ${𝒟}_{u}=\{x_{i}{\}}_{i=1}^{N}$.
*N*	The total number of samples in the training dataset.
$x,x_{i}$	An input sample (e.g., image) from the dataset.
𝒲(·)	The weak data augmentation function.
𝒜(·)	The strong data augmentation function.
***Network Architecture***	
f(Θ;·)	The shared feature extractor (Encoder) parameterized by Θ.
*z*	The latent feature representation vector, z=f(Θ;x).
$g_{clu}(\theta_{clu};\cdot )$	The Clustering Head parameterized by $\theta_{clu}$, mapping features to class logits.
$h_{\theta }$	The frozen Teacher network in the PEC module with fixed random parameters θ.
$g_{\phi^{c}}$	The Student network specific to cluster *c* with trainable parameters $\phi^{c}$.
σ(·)	The Softmax activation function.
***PEC Confidence Mechanism***	
*C*	The total number of pre-defined clusters (or classes).
$E_{c}(x)$	The prediction error of sample *x* regarding cluster *c* (squared Euclidean distance).
$conf_{c}(x)$	The calibrated confidence score for cluster *c* derived from prediction error.
α	The scaling hyperparameter controlling the sensitivity of confidence conversion.
${\tilde{p}}^{pec}(c|x)$	The normalized cluster probability distribution derived from PEC scores.
*mconf*(*x*)	The maximum confidence score for sample *x*, defined as $\max_{c}{\tilde{p}}^{pec}(c|x)$.
***Dynamic Selection Strategy***	
$\hat{y}(x)$	The tentative pseudo-label assigned to sample *x* based on PEC probability.
${𝒯}_{c}$	The candidate set of samples tentatively assigned to cluster *c*.
*K*	Hyperparameter denoting the maximum candidate capacity per cluster.
*M*(*c*)	The dynamic sample capacity (core sample size) calculated for cluster *c*.
${𝒮}_{c}$	The subset of high-quality pseudo-labeled samples selected for cluster *c*.
𝒮	The final set of reliable pseudo-labeled pairs used for training, $𝒮=\{(x,\hat{y})\}$.
***Optimization and Training***	
${ℒ}_{clu}$	The semantic clustering loss (Cross-Entropy) for the Clustering Head.
${ℒ}_{pec}$	The calibration loss (Mean Squared Error) for updating PEC Student networks.
${𝐩}_{i}^{\text{clu}}$	The probability vector predicted by the Clustering Head for sample $x_{i}$.
${𝐳}_{sg}$	The latent feature representation with the stop-gradient operator applied.
sg(·)	The stop-gradient operator used to decouple feature learning from regression.
*B*	The batch size used for mini-batch optimization.
***Inference***	
$y^{pred}$	The final predicted class label for a test sample.

### Prediction Error-based Classification (PEC)

The Prediction Error-based Classification (PEC) framework [20] adopts a regression-based paradigm to model class distributions. Unlike traditional discriminative approaches that optimize decision boundaries between classes, PEC utilizes a teacher-student architecture to estimate class membership via regression consistency.

### Network architecture and training

The framework comprises two distinct components: a shared teacher network and a set of class-specific student networks. The teacher network, denoted as $h_{\theta }:{\mathbb{R}}^{n}\to {\mathbb{R}}^{d}$, maps inputs *x* to a *d*-dimensional embedding space. A critical characteristic of PEC is that the parameters θ are randomly initialized and remain *frozen* throughout the entire training process.

For each class c∈{1,…,C}, a distinct student network $g_{\phi^{c}}$ is instantiated with learnable parameters $\phi^{c}$. During the training phase, the student $g_{\phi^{c}}$ is optimized to replicate the output of the fixed teacher network $h_{\theta }$ using exclusively the data $X_{c}$ belonging to class *c*. The objective function minimizes the Mean Squared Error (MSE) between the prediction of the student and the target output of the teacher.

### Inference and Working Principle

A student network $g_{\phi^{c}}$ effectively learns to approximate the random mapping of the teacher on the in-distribution data manifold (i.e., samples from class *c*). Consequently, the regression error $\left\|g_{\phi^{c}}(x)-h_{\theta }(x)\right\|$ is expected to be minimal for an input *x* belonging to class *c*. Conversely, for out-of-distribution inputs (i.e., samples from classes k≠c), the student fails to generalize, resulting in a significantly higher prediction error. During inference, a test sample *x* is processed simultaneously by the frozen teacher and all trained student networks. The classification decision is determined by identifying the class index corresponding to the student with the minimal prediction error, calculated as follows [20]:


$$\begin{array}{c} y^{pred}={\text{arg}\min}_{c\in \{1,\ldots ,C\}}\|g_{\phi^{c}}(x)-h_{\theta }(x)\|. \end{array}$$ (1)

### Calibrated Deep Clustering (CDC)

The Calibrated Deep Clustering (CDC) method [15] addresses the prevalent overconfidence issue in deep clustering, where the estimated confidence of a model significantly exceeds its actual prediction accuracy. Unlike conventional clustering approaches that rely solely on pseudo-labels with fixed thresholds, CDC introduces a dual-head framework comprising a Clustering Head and a Calibration Head to simultaneously achieve high clustering accuracy and well-calibrated confidence estimates.

### Data augmentation and feature extraction

Formally, let ${𝒟}_{u}=\{x_{i}:i\in \{1,2,\ldots ,N\}\}$ denote a training set of *N* unlabeled samples. To learn robust representations, the framework employs distinct augmentation strategies utilized in [6]: a weak augmentation 𝒲(·) and a strong augmentation 𝒜(·). For each sample $x_{i}$, these strategies generate a weakly augmented view $𝒲(x_{i})$ and a strongly augmented view $𝒜(x_{i})$, respectively.

Let f(Θ;·) be the shared feature extractor. The augmented views are fed into f(Θ;·) to extract latent features. Specifically, the feature embedding for a given view is obtained as $z_{i}=f(\Theta ;{\tilde{x}}_{i})$, where ${\tilde{x}}_{i}\in \{𝒲(x_{i}),𝒜(x_{i})\}$. These embeddings $z_{i}$ serve as the input for the subsequent dual-head modules.

### Dual-head calibration mechanism

The extracted features are processed by two parallel heads: the Clustering Head, denoted as $g_{clu}(\theta_{clu};\cdot )$, and the Calibration Head, denoted as $g_{cal}(\theta_{cal};\cdot )$. Both heads utilize the softmax function σ(·) to map the features to a probability distribution over *C* predetermined classes.

The core innovation of CDC lies in its calibration strategy, which posits that reliable predictions should exhibit local consistency in the feature space. To implement this, the feature space is partitioned into *K* mini-clusters using the K-means algorithm. For each mini-cluster $Q_{k}$, the target distribution ${\hat{q}}_{k}$ is derived by averaging the predictions of the Clustering Head for all samples within that region:


$$\begin{array}{c} {\hat{q}}_{k}=\frac{1}{\left|Q_{k}\right|}\sum_{x_{i}\in Q_{k}}\sigma (g_{clu}(\theta_{clu};f(\Theta ;x_{i}))), \end{array}$$ (2)

where $\left|Q_{k}\right|$ denotes the number of samples in the mini-cluster.

The Calibration Head is then optimized to align its pixel-wise confidence with this regionally smoothed target. The calibration loss ${ℒ}_{cal}$ minimizes the divergence between the output of the Calibration Head and the mini-cluster average target:


$$\begin{array}{c} {ℒ}_{cal}=-\frac{1}{B}\sum_{k}\sum_{x_{i}\in Q_{k}}{\hat{q}}_{k}\log(\sigma (g_{cal}(\theta_{cal};f(\Theta ;x_{i})))), \end{array}$$ (3)

where *B* is the batch size. This joint optimization allows the Calibration Head to estimate realistic confidence scores, which are subsequently used by the Clustering Head to dynamically select reliable pseudo-labels.

### Dynamic selection and clustering optimization

Leveraging the calibrated confidence from the Calibration Head, CDC adopts a dynamic thresholding strategy to select high-quality samples for updating the Clustering Head. Specifically, for each class *c*, a dynamic sample capacity *M*(*c*) is calculated by aggregating the confidence of weakly augmented samples estimated by the Calibration Head. The top-*M*(*c*) samples with the highest confidence in each class are selected to form the pseudo-label set 𝒮.

Finally, the Clustering Head and the shared feature extractor are optimized using the standard Cross-Entropy loss on the strongly augmented views of these selected samples:


$$\begin{array}{c} {ℒ}_{clu}=-\frac{1}{\left|𝒮\right|}\sum_{(x_{i},y_{i})\in 𝒮}\log(\sigma (g_{clu}(\theta_{clu};f(\Theta ;𝒜(x_{i}))){)}_{y_{i}}), \end{array}$$ (4)

where $y_{i}$ is the pseudo-label assigned by the calibration process. This step ensures that the clustering head learns semantic representations from reliable regions while avoiding the confirmation bias typically caused by fixed global thresholds.

### PEC-CDC framework

In this study, we propose PEC-CDC, a novel framework that reconfigures the architectural paradigm of Calibrated Deep Clustering (CDC). While the original CDC framework comprises a shared feature extractor f(Θ;·), a Clustering Head ($g_{clu}$), and a Calibration Head ($g_{cal}$), our approach replaces the conventional probability-based $g_{cal}$ with a regression-based PEC Head.

This architectural evolution addresses the critical need for a reliable, absolute metric for confidence estimation. Conventional softmax-based calibration often suffers from inherent overconfidence due to relative normalization, assigning high probabilities even to ambiguous samples that lack discriminative features. To address this limitation, the PEC Head introduces a frozen random teacher network and a set of cluster-specific student networks. By training students to regress the fixed output of the teacher, we derive an absolute prediction error that serves as a direct metric for distributional support. This error-based confidence is subsequently employed to dynamically regulate pseudo-label selection, ensuring that the self-training process is guided by intrinsic uncertainty rather than relative logits.

### Architecture

Structurally, the PEC-CDC framework retains the shared feature extractor f(Θ;·) and the Clustering Head $g_{clu}$ to preserve the model capability for learning discriminative semantic representations. The core modification lies in the functional replacement of the Calibration Head $g_{cal}$ with the PEC Head.

**Fig 1:**
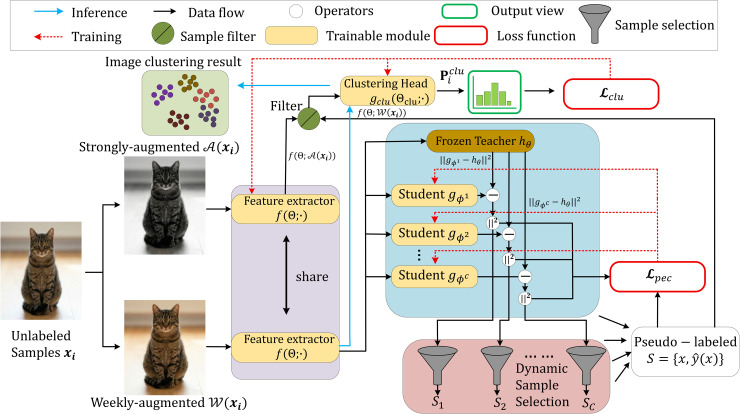
Overview of the PEC-CDC framework. The architecture operates through dual pathways: 1) The **Semantic Stream** (top) utilizes strongly augmented views $𝒜(x_{i})$ to optimize the Clustering Head $g_{clu}$ via the semantic consistency loss ${ℒ}_{clu}$. 2) The **Calibration Stream** (bottom) employs weakly augmented views $𝒲(x_{i})$ to drive the PEC Head. Within the PEC Head, a set of cluster-specific student networks $g_{\phi^{c}}$ learn to regress the output of a frozen teacher network $h_{\theta }$. This process generates absolute prediction errors, which serve as a reliable metric for distributional uncertainty to guide the Dynamic Sample Selection (DSS) mechanism. Finally, high-quality pseudo-labels filtered by DSS supervise the training of the Clustering Head.

In this new configuration, the PEC Head assumes the role of confidence estimator but operates on a fundamentally different principle: absolute prediction error rather than relative softmax probability. As illustrated in Fig 1, the PEC Head processes the latent features *z* generated by the encoder and consists of two key components:

1. **Frozen Teacher (**$h_{\theta }$**):** A randomly initialized network with fixed weights θ. It defines a complex but static function space that serves as the regression target for the latent features.
2. **Cluster-Specific Students (**$g_{\phi^{c}}$**):** A set of learnable networks parameterized by $\phi^{c}$, where the *c*-th student is trained exclusively to regress the output of the teacher for samples belonging to cluster *c*.

By replacing the single $g_{cal}$ with this Teacher-Student module, the framework shifts from optimizing a calibration loss to minimizing the regression error of the students. Consequently, the source of confidence for the dynamic sample selection mechanism is updated from calibrated softmax scores to the inverse of the prediction errors of the students.

To integrate the regression-based PEC module with the dynamic thresholding mechanism of CDC, we first quantify the absolute uncertainty of a sample. For a given sample *x*, let z=f(Θ;x) denote its latent feature representation. The prediction error regarding cluster *c*, denoted as $E_{c}(x)$, is calculated as the squared Euclidean distance between the student and teacher outputs:


$$\begin{array}{c} E_{c}(x)=\|g_{\phi^{c}}(z)-h_{\theta }(z){\|}^{2}. \end{array}$$ (5)

Subsequently, we convert this error profile into a confidence metric. The Calibrated Confidence Score for cluster *c*, denoted as $conf_{c}(x)$, is derived via an exponential transformation of the prediction error:


$$\begin{array}{c} conf_{c}(x)=\exp(-\alpha E_{c}(x)), \end{array}$$ (6)

where α is a scaling hyperparameter controlling the sensitivity to errors.

Finally, to generate a probabilistic output compatible with the selection strategy, we normalize these confidence scores to obtain the Cluster Probability, denoted as ${\tilde{p}}^{pec}(c|x)$:


$$\begin{array}{c} {\tilde{p}}^{pec}(c|x)=\frac{conf_{c}(x)}{\sum_{j=1}^{C}conf_{j}(x)}. \end{array}$$ (7)

Structurally, this mechanism replaces the confidence source of the CDC framework without altering its overall self-training backbone. Unlike standard methods that apply softmax directly to the CluHead logits, our approach derives probabilities strictly from regression consistency. By measuring how well the student network mimics the output of the teacher (where lower error signifies higher distributional consistency), ${\tilde{p}}^{pec}(c|x)$ avoids the issue of overconfidence typically observed with direct logit-based probabilities, particularly during the early stages of training. The following subsection further explains the theoretical rationale behind using prediction error as a reliability signal for sample–cluster consistency and pseudo-label selection.

### Theoretical link between prediction error and clustering reliability

Since PEC-CDC uses prediction error as the basis of confidence estimation, the key question is why a smaller cluster-specific regression error can indicate stronger sample–cluster consistency and more reliable pseudo-labels. The reliability of prediction error in PEC-CDC can be understood from the teacher–student mechanism adopted in PEC. In PEC, class-specific student networks are trained to approximate a frozen teacher network, and the class with the smallest student–teacher prediction error is selected [20]. When this idea is incorporated into the CDC-style self-training pipeline, prediction error can serve as a confidence-related signal for selecting reliable pseudo-labels, which is consistent with the role of calibrated confidence in CDC [15].

Let $z=f_{\Theta }(x)$ denote the latent representation of sample *x*, and let ${ℳ}_{c}$ denote the latent support of cluster *c*. In the PEC module, the frozen teacher $h_{\theta }$ provides a fixed target mapping, and each cluster-specific student $g_{\phi^{c}}$ is trained using samples assigned to cluster *c*. The prediction error of *x* with respect to cluster *c* is defined as


$$\begin{array}{c} E_{c}(x)={\left\|g_{\phi^{c}}(z)-h_{\theta }(z)\right\|}^{2}. \end{array}$$ (8)

We assume that the samples used to train the *c*-th student are dominated by samples from ${ℳ}_{c}$, with bounded contamination from other clusters. Under this condition, the student $g_{\phi^{c}}$ is expected to approximate the teacher more accurately on ${ℳ}_{c}$ than on regions far from ${ℳ}_{c}$. Formally, for constants $\epsilon_{in}<\epsilon_{out}$, we assume


$$\begin{array}{c} 𝔼\left[E_{c}(x)|z\in {ℳ}_{c}\right]\leq \epsilon_{in},\;𝔼\left[E_{c}(x)|z\notin {𝒩}_{\rho }({ℳ}_{c})\right]\geq \epsilon_{out}, \end{array}$$ (9)

where ${𝒩}_{\rho }({ℳ}_{c})$ denotes a ρ-neighborhood of ${ℳ}_{c}$.

Under this approximation assumption, a lower $E_{c}(x)$ indicates that sample *x* is more consistent with the distributional structure learned by the *c*-th student. For a tentative pseudo-label $\hat{y}$, if


$$\begin{array}{c} E_{\hat{y}}(x)+\Delta <E_{k}(x),\;\forall k\neq \hat{y}, \end{array}$$ (10)

where Δ>0 is an error margin, then the selected cluster provides stronger distributional support for *x* than the alternatives. Under this condition, the pseudo-label $\hat{y}$ can be regarded as more reliable. In contrast, noisy samples, samples misassigned at early stages, or samples near ambiguous cluster boundaries are usually not well fitted by a single cluster-specific student. They tend to produce larger errors or smaller error margins, making them less likely to be selected as reliable pseudo-labels.

PEC-CDC converts prediction error into an error-based confidence score through a monotonic decreasing function,


$$\begin{array}{c} conf_{c}(x)=\exp\left(-\alpha E_{c}(x)\right), \end{array}$$ (11)

where α>0. For a fixed cluster *c*, $conf_{c}(x)$ decreases monotonically as $E_{c}(x)$ increases, so ranking samples within the same cluster by $conf_{c}(x)$ is equivalent to ranking them by prediction error in the opposite direction. Moreover, because the subsequent normalization is applied to positive confidence scores, the selected cluster for a given sample is still the one with the smallest prediction error, and a larger error margin generally leads to a higher normalized confidence. This provides a direct link between prediction error and the dynamic selection of reliable pseudo-labels in PEC-CDC.

This analysis also explains the difference between PEC-CDC and probability-based calibration. Softmax confidence reflects relative preference among clusters, while prediction error evaluates whether a sample conforms to the distributional structure learned by a cluster-specific student. This is consistent with the theoretical motivation of PEC, which relates prediction error to uncertainty through Gaussian Process posterior variance [20]. Therefore, within the CDC-style self-training pipeline [15], prediction error serves as an assumption-based reliability signal for sample–cluster consistency and pseudo-label selection.

### Training workflow

The training process leverages the PEC Head to guide the Clustering Head through a rigorous dynamic pseudo-label selection mechanism. This workflow ensures that pseudo-labels are generated based on the structural consistency validated by the regression module. Based on the above reliability rationale, samples with lower cluster-specific prediction errors are assigned higher confidence and are preferentially retained during dynamic sample selection. The optimization proceeds in two coordinated steps:

### Phase 1: Dynamic sample selection

In each epoch, we utilize the PEC-derived confidence to determine which samples are reliable enough to serve as pseudo-labels. The selection process proceeds in three stages for each cluster *c*:

1. **Assignment:** First, we assign a tentative pseudo-label $\hat{y}(x)$ to each sample based on the maximum PEC probability and calculate its associated confidence score conf(*x*):

$$\begin{array}{c} \hat{y}(x)={\text{arg}\max}_{c\in \{1,\ldots ,C\}}{\tilde{p}}^{pec}(c|x),\;mconf(x)=\max_{c\in \{1,\ldots ,C\}}{\tilde{p}}^{pec}(c|x). \end{array}$$ (12)

Here, conf(*x*) serves as a normalized confidence metric lying within the range [0, 1]. It is important to note that this inference step enforces a hard assignment, meaning each sample is tentatively allocated to exactly one cluster—specifically, the one maximizing the calibrated probability.

2. **Capacity Estimation and Pseudo-Label Generation:** We adopt a dynamic budgeting strategy to determine the number of reliable samples to retain for each cluster. First, we collect all samples tentatively assigned to cluster *c* (i.e., $\left\{x|\hat{y}(x)=c\right\}$) and sort them in descending order based on their confidence scores *mconf*(*x*). From this sorted sequence for each specific cluster *c*, we retain the top-*K* ranked samples to form the candidate set ${𝒯}_{c}$, where *K* is a pre-defined hyperparameter acting as the maximum capacity limit.

To adapt to the varying difficulty and distribution density of different clusters, we calculate a specific core sample size *M*(*c*) for each cluster *c* by aggregating the PEC-derived confidence scores of these candidate samples:


$$\begin{array}{c} M(c)=\left\lfloor \sum_{x\in {𝒯}_{c}}mconf(x)\right\rfloor . \end{array}$$ (13)

This formulation interprets the cumulative confidence mass as a proxy for the effective cluster capacity, thereby enabling the dynamic sample capacity *M*(*c*) to adaptively scale according to the reliability of each cluster, in contrast to global thresholds. Guided by the dynamic sample capacity *M*(*c*), we construct the pseudo-labeled sample set ${𝒮}_{c}$ by performing a secondary selection on the candidate set ${𝒯}_{c}$. Since ${𝒯}_{c}$ is already sorted by confidence, we explicitly define the pseudo-labeled sample set ${𝒮}_{c}$ in Cluster *c* by imposing a rank-based constraint to retain strictly the first *M*(*c*) samples:


$$\begin{array}{c} {𝒮}_{c}=\left\{x_{i}\in {𝒯}_{c}|1\leq i\leq M(c)\right\}, \end{array}$$ (14)

where *i* denotes the ranking index of the sample within the sorted candidate set ${𝒯}_{c}$. The final pseudo-labeled samples is defined as:


$$\begin{array}{c} 𝒮=\left\{(x,\hat{y}(x))|x\in {𝒮}_{c},c=1,...,C\right\}. \end{array}$$ (15)

This strategy utilizes the PEC module as an independent evaluator to mitigate the noise inherent in self-training. Given that the confidence score conf(*x*) is inversely correlated with the regression error, clusters characterized by lower aggregate errors imply better-learned distributions. Consequently, these reliable clusters naturally yield a larger core sample size *M*(*c*) and retain a greater number of samples. In contrast, ambiguous clusters associated with higher regression errors result in a reduced selection core sample size, which effectively prevents the accumulation of noisy pseudo-labels.

### Phase 2: Decoupled optimization

Once the reliable pseudo-label set 𝒮 is constructed, we proceed to update the network parameters. To ensure training stability and prevent the regression task from interfering with semantic feature learning, we adopt a decoupled optimization strategy. This phase consists of two distinct update steps performed within the same epoch:

1. **Clustering Optimization (Semantic Learning):** The primary goal of this step is to enforce semantic consistency by encouraging the model to predict the generated pseudo-labels confidently. We utilize the strongly augmented view $𝒜(x_{i})$ as input to enhance representation robustness.

Let $𝒮=\{(x_{i},{\hat{y}}_{i}){\}}_{i=1}^{\left|𝒮\right|}$ be the selected labeled set. For each sample $x_{i}$, let ${𝐩}_{i}^{\text{clu}}=\sigma (g(\theta_{clu};f(\Theta ;𝒜(x_{i}))))\in {\mathbb{R}}^{C}$ denote the predicted probability distribution, and let ${𝐲}_{i}$ be the one-hot vector representation of the pseudo-label ${\hat{y}}_{i}$. The objective is minimized via the Cross-Entropy loss:


$$\begin{array}{c} {ℒ}_{clu}=-\frac{1}{\left|𝒮\right|}\sum_{(x_{i},{\hat{y}}_{i})\in S}{\hat{y}}_{i}\log({𝐩}_{i}^{\text{clu}}), \end{array}$$ (16)

2. **PEC Optimization (Calibration Update):** Simultaneously, we update the PEC module to maintain accurate confidence estimation. We utilize the weakly augmented view 𝒲(x) to reduce distribution shift. To prevent the regression objective from distorting the semantic feature space learned by the encoder, we apply the stop-gradient operation on the feature input.

Specifically, the **Cluster-Specific Students** $g_{\phi^{c}}$ are updated to minimize the Mean Squared Error (MSE) between their predictions and the outputs of the **Frozen Teacher** $h_{\theta }$. Adopting a class-wise aggregation, the loss function is defined as:


$$\begin{array}{c} {ℒ}_{pec}=\sum_{c=1}^{C}{𝔼}_{x:\hat{y}=c}{\left\|g_{\phi^{c}}\left({𝐳}_{sg}\right)-h_{\theta }\left({𝐳}_{sg}\right)\right\|}^{2}, \end{array}$$ (17)

where ${𝐳}_{sg}=\text{sg}(f(\Theta ;𝒲(x)))$ denotes the feature representation with the stop-gradient operator sg(·) applied. Here, *C* is the total number of classes, and ${𝔼}_{x:\hat{y}=c}$ denotes the expectation over samples assigned to cluster *c*. This objective ensures that each student $g_{\phi^{c}}$ specifically fits the regression manifold defined by the static teacher $h_{\theta }$ within its corresponding cluster.

**Algorithm 1 PEC-CDC: Deep Clustering with PEC-based Confidence**

**Require:** Unlabeled dataset ${𝒟}_{u}$; Number of clusters *C*; Max epochs $E_{max}$; Scaling parameter α.

**Require:** Weak augmentation 𝒲(·) and strong augmentation 𝒜(·).

**Require:** Candidate set size *K* (Maximum capacity limit).

1:  **Initialize Backbone:** Shared Feature Extractor f(Θ) (e.g., MoCo-v2 pre-trained), Clustering Head $g_{clu}(\theta_{clu})$.

2:  **Initialize PEC Module:**

3:   Frozen Teacher $h_{\theta }$ (Randomly initialized, fixed weights).

4:    Cluster-Specific Students $\{g_{\phi^{c}}{\}}_{c=1}^{C}$ (Randomly initialized, learnable).

5:  **for** epoch = 1 to $E_{max}$ **do**

6:   **// Phase 1: Dynamic Sample Selection (Global or Batch-wise)**

7:   Extract weak features ${𝒵}^{w}=\{z_{i}=f(\Theta ;𝒲(x_{i}))|x_{i}\in {𝒟}_{u}\}$

8:   Initialize candidate sets ${𝒯}_{c}=\emptyset$ for c=1…C

9:                     ▷ *1. Confidence Estimation & Assignment*

10:  **for** each sample $x_{i}\in {𝒟}_{u}$ **do**

11:   **for** *c* = 1 to *C* **do**

12:     $E_{c}(x_{i})\leftarrow \|g_{\phi^{c}}(z_{i})-h_{\theta }(z_{i}){\|}^{2}$        ▷ Prediction Error (Eq. 5)

13:     $conf_{c}(x_{i})\leftarrow \exp(-\alpha E_{c}(x_{i}))$

14:   **end for**

15:   ${\tilde{p}}^{pec}(c|x_{i})\leftarrow \frac{conf_{c}(x_{i})}{\sum_{j}conf_{j}(x_{i})}$         ▷ PEC Probability (Eq. 7)

16:   ${\hat{y}}_{i}\leftarrow \text{arg}\max_{c}{\tilde{p}}^{pec}(c|x_{i})$; $mconf(x_{i})\leftarrow \max_{c}{\tilde{p}}^{pec}(c|x_{i})$

17:   Add $\left(x_{i},mconf(x_{i})\right)$ to tentative set ${ℐ}_{{\hat{y}}_{i}}$

18:  **end for**

19:               ▷ *2. Capacity Estimation & Pseudo-Label Generation*

20:  **for** *c* = 1 to *C* **do**

21:   Sort ${ℐ}_{c}$ by confidence conf(*x*) in descending order

22:    ${𝒯}_{c}\leftarrow$ Top-*K* samples from ${ℐ}_{c}$ ▷ Form Candidate Set

23:    $M(c)\leftarrow \lfloor \sum_{x\in {𝒯}_{c}}\text{conf}(x)\rfloor$        ▷ Calculate Dynamic Sample Capacity (Eq. 13)

24:    ${𝒮}_{c}\leftarrow$ Top-*M*(*c*) samples from ${𝒯}_{c}$ ▷ Final Selection

25:   **end for**

26:  $𝒮\leftarrow {⋃}_{c=1}^{C}\{(x,c)|x\in {𝒮}_{c}\}$         ▷ Construct Pseudo-label Set (Eq. 15)

27:  **// Phase 2: Decoupled Optimization**

28:  **for** each minibatch ℬ⊂𝒮 **do**

29:             ▷ *Step 2.1: Clustering Optimization (Semantic Learning)*

30:   Get strong views: $x^{s}\leftarrow 𝒜(x)$ for $(x,\hat{y})\in ℬ$

31:    Compute logits: ${𝐩}^{clu}\leftarrow \sigma (g_{clu}(\theta_{clu};f(\Theta ;x^{s})))$

32:    ${ℒ}_{clu}\leftarrow -\frac{1}{\left|ℬ\right|}\sum_{(x,\hat{y})\in ℬ}{\hat{y}}_{i}\log({𝐩}_{i}^{\text{clu}})$ ▷ Eq. 16

33:   Update $\Theta ,\theta_{clu}$ to minimize ${ℒ}_{clu}$

34:                ▷ *Step 2.2: PEC Optimization (Calibration Update)*

35:   Get weak views: $x^{w}\leftarrow 𝒲(x)$ for $(x,\hat{y})\in ℬ$

36:    ${𝐳}_{sg}\leftarrow \text{sg}(f(\Theta ;x^{w}))$ ▷ Stop-Gradient on Encoder

37:    ${ℒ}_{pec}\leftarrow \sum_{c=1}^{C}{𝔼}_{(x,\hat{y})\in ℬ:\hat{y}=c}{\left\|g_{\phi^{c}}({𝐳}_{sg})-h_{\theta }({𝐳}_{sg})\right\|}^{2}$ ▷ Eq. 17

38:    Update PEC students $\{\phi^{c}{\}}_{c=1}^{C}$ to minimize ${ℒ}_{pec}$

39:   **end for**

40: **end for**

### Algorithmic overview

**Algorithm 1** outlines the comprehensive training procedure of the proposed PEC-CDC framework. Lines 4–16 execute the global dynamic sample selection phase at the onset of each epoch, where the PEC module evaluates all unlabeled samples to compute the regression-based confidence and determine the per-cluster dynamic quota *M*(*c*). Based on this global rank, the reliable pseudo-labeled set 𝒮 is constructed to guide the subsequent training. The decoupled optimization strategy is then performed in Lines 18–26 within the mini-batch loop. Specifically, Lines 20–22 optimize the Clustering Head using the semantic consistency loss ${ℒ}_{clu}$ on strongly augmented views, while Lines 24–26 simultaneously update the PEC students via the calibration loss ${ℒ}_{pec}$ on weakly augmented views. Notably, the stop-gradient operation in Line 25 ensures that the regression objective improves confidence estimation without interfering with the semantic representation learning of the encoder.

Upon the completion of training, the Clustering Head $g_{clu}$ has established discriminative semantic boundaries under the supervision of the high-quality pseudo-labels filtered by the PEC module. Although the PEC module serves as an effective calibrator for selecting reliable samples during the training phase, the Clustering Head is directly optimized for class separation and semantic robustness. Therefore, we utilize the Clustering Head to make the final prediction for the *i*-th sample $x_{i}$, computed as $y_{i}^{pred}=\text{arg}\max\sigma (g_{clu}(\theta_{clu};f(\Theta ;x_{i})))$.

### Computational complexity analysis

We further analyze the computational complexity of PEC-CDC to clarify its scalability relative to the baseline CDC framework. Let *N* denote the number of unlabeled samples, *C* denote the number of clusters, and |𝒮| denote the number of selected pseudo-labeled samples in each epoch. We denote the per-sample computational costs associated with the encoder, Clustering Head, frozen teacher, and one cluster-specific student network as $T_{f}$, $T_{clu}$, $T_{t}$, and $T_{s}$, respectively. These terms abstract the corresponding forward or optimization costs required by each stage.

For time complexity, PEC-CDC follows the same self-training backbone as CDC and mainly differs in the confidence estimation stage. In each epoch, weakly augmented samples are first passed through the encoder, requiring $O(NT_{f})$. Then, the PEC module evaluates the prediction error of each sample with respect to all *C* cluster-specific students. Since the teacher is shared and fixed, this step requires $O(NT_{t}+NCT_{s})$ time, together with *O*(*NC*) operations for error-to-confidence conversion and normalization. The dynamic sample selection step sorts samples within clusters according to PEC-derived confidence. Its worst-case cost is O(NlogN), and the practical cost can be reduced by partial top-*K* selection. After the reliable set 𝒮 is obtained, clustering optimization requires $O(|𝒮|(T_{f}+T_{clu}))$, while PEC optimization requires $O(|𝒮|(T_{f}+T_{t}+T_{s}))$ because each selected sample updates only the student corresponding to its assigned cluster. Therefore, the overall per-epoch time complexity of PEC-CDC can be written as


$$\begin{array}{c} O\left(NT_{f}+NT_{t}+NCT_{s}+NC+N\log N+|𝒮|(T_{f}+T_{clu}+T_{t}+T_{s})\right). \end{array}$$ (18)

Compared with CDC, PEC-CDC replaces the probability-based Calibration Head with the PEC module. Thus, the additional time cost mainly comes from evaluating *C* lightweight student networks during confidence estimation. Importantly, this overhead is linear in both *N* and *C*, and the method does not require pairwise sample comparisons or global graph construction with *O*(*N*^2^) complexity. Since *C* is fixed by the number of target clusters and the student networks are lightweight heads, the practical overhead remains moderate. This is consistent with the design goal of preserving the scalability of the CDC self-training pipeline while improving the reliability of confidence estimation.

For space complexity, PEC-CDC stores the shared encoder, the Clustering Head, the frozen teacher, and *C* cluster-specific student networks. Let $P_{f}$, $P_{clu}$, $P_{t}$, and $P_{s}$ denote the numbers of parameters of these components, respectively. The parameter memory of PEC-CDC is therefore


$$\begin{array}{c} O(P_{f}+P_{clu}+P_{t}+CP_{s}). \end{array}$$ (19)

Compared with CDC, whose additional calibration parameters are $P_{cal}$, PEC-CDC replaces the original calibration-side parameter storage with $P_{t}+CP_{s}$. Therefore, the net additional parameter cost is approximately $P_{t}+CP_{s}-P_{cal}$. Since the teacher is frozen and the student networks are implemented as lightweight modules, this additional parameter cost is limited. During dynamic sample selection, PEC-CDC only needs to store tentative labels, confidence scores, and candidate sets, leading to *O*(*N* + *CK*) memory when confidence scores are computed in a streaming or mini-batch manner. In the worst case, storing all sample-cluster confidence scores requires *O*(*NC*) memory. Therefore, PEC-CDC avoids quadratic memory growth with respect to *N* and remains feasible for large-scale deep clustering tasks.

## Experimental results and analysis

### Linear evaluation

Following standard evaluation protocols in unsupervised representation learning, we assess the quality of learned representations by training a linear classifier on frozen features extracted by the encoder. Experiments were conducted on four benchmark datasets: CIFAR-10 [42], CIFAR-100 [42], ImageNet-100 [43], and EuroSAT [44].

The linear classifier is trained on frozen representations using Stochastic Gradient Descent (SGD). We report Top-1 and Top-5 classification accuracies on the test sets. We compare our proposed method with a comprehensive set of state-of-the-art self-supervised learning methods. Baselines include contrastive learning approaches such as SimCLR [30], DINO [32], MoCo-v2 [29], and MoCo-v3 [31]; clustering-based methods such as SwAV [9]; redundancy reduction methods such as Barlow Twins (BT) [35] and VICReg [36]; and recent hybrid approaches such as BYOL [33] and CueCo [7]. Baseline results are sourced from [7].

Quantitative comparison results are summarized in Table 3. Overall, PEC-CDC achieves competitive or superior performance across the evaluated datasets, indicating that the proposed prediction error-based calibration mechanism can improve representation quality while preserving strong generalization ability.

**Table 3:** Linear evaluation results (Top-1 and Top-5 accuracy %) on CIFAR-10, CIFAR-100, and ImageNet-100 using a ResNet-18 backbone. The methods are listed in the specified order, with our method at the bottom. Best results are highlighted in bold, and second-best results are underlined.

Method	CIFAR-10		CIFAR-100		ImageNet-100	
	Top-1	Top-5	Top-1	Top-5	Top-1	Top-5
BYOL [33]	92.61	99.82	**70.18**	91.36	80.09	94.99
DINO [32]	89.19	99.31	66.38	90.18	74.84	92.92
SimSiam [34]	90.51	99.79	65.86	89.48	77.04	94.02
MoCo-v2 [29]	92.94	99.79	69.54	**91.49**	78.20	95.50
MoCo-v3 [31]	93.10	**99.90**	68.83	90.57	80.86	95.18
ReSSL [45]	90.63	99.62	65.83	89.51	76.59	94.41
VICReg [36]	90.07	99.71	68.54	90.83	79.22	95.06
SwAV [9]	89.17	99.68	64.67	88.52	74.28	92.84
SimCLR [30]	90.74	99.75	65.39	88.58	77.48	93.42
BT [35]	89.57	99.73	69.18	91.19	78.62	94.72
CueCo [7]	91.40	99.79	68.56	91.05	78.65	94.03
**OUR**	**94.05**	99.78	70.07	91.27	**81.80**	**95.60**

On CIFAR-10, our method achieves a Top-1 accuracy of **94.05%**, significantly outperforming all competing baselines. Notably, it surpasses the strong MoCo-v3 baseline (93.10%) and BYOL (92.61%). This demonstrates the effectiveness of our approach in learning discriminative features from relatively small-scale images, successfully mitigating feature collapse. For the more challenging CIFAR-100 dataset, involving fine-grained classification, our method shows high robustness with a Top-1 accuracy of **70.07%**. This performance is comparable to the leading method BYOL (70.18%) and surpasses prominent baselines such as MoCo-v2 (69.54%), VICReg (68.54%), and CueCo (68.56%). These results suggest that our framework effectively captures subtle semantic differences between classes. On the larger-scale ImageNet-100, our method exhibits superior scalability, achieving the highest Top-1 accuracy of **81.80%** and Top-5 accuracy of **95.60%** among all compared methods. This represents a substantial improvement over MoCo-v3 (80.86%) and BYOL (80.09%). The consistent gains on ImageNet-100 validate the generalization capability of our framework, confirming its effectiveness in handling diverse visual data compared to existing approaches.

To further examine the generalization ability of PEC-CDC beyond standard natural image benchmarks, we additionally evaluated the learned representations on the EuroSAT dataset. EuroSAT is constructed from Sentinel-2 satellite images and contains 27,000 labeled and geo-referenced images from 10 land use and land cover classes [44]. Compared with CIFAR and ImageNet-style datasets, EuroSAT represents a remote sensing scenario with different visual patterns and spatial structures, providing a useful benchmark for evaluating cross-domain representation quality. Since not all baselines provided available EuroSAT results under the same setting, we compared PEC-CDC with several representative methods selected from Table 3, including BYOL [33], DINO [32], SimSiam [34], MoCo-v2 [29], MoCo-v3 [31], and SimCLR [30]. The results are summarized in Table 4.

**Table 4:** Linear evaluation results on EuroSAT using a ResNet-18 backbone. Top-1 and Top-5 accuracies are reported.

Metric	BYOL	DINO	SimSiam	MoCo-v2	MoCo-v3	SimCLR	PEC-CDC
Top-1	92.18	92.70	90.74	89.67	91.48	89.44	**94.67**
Top-5	99.77	99.75	99.79	**99.96**	99.85	99.57	**99.96**

As shown in Table 4, PEC-CDC achieves the best Top-1 accuracy of 94.67% on EuroSAT, outperforming all compared baselines. Specifically, it improves Top-1 accuracy by 1.97 percentage points over DINO and by 2.49 percentage points over BYOL. PEC-CDC also obtains a Top-5 accuracy of 99.96%, matching the best result among the compared methods. These results indicate that the learned representations remain discriminative in the satellite imaging domain, where image characteristics differ from standard natural images. This further suggests that PEC-CDC has practical applicability beyond standard object-centered benchmarks.

In summary, the linear evaluation results show that PEC-CDC learns effective and transferable representations across standard object-centered datasets, large-scale visual benchmarks, and the EuroSAT satellite imaging dataset. The consistent improvement or competitive performance across these settings indicates that the proposed prediction error-based calibration mechanism can enhance representation learning without sacrificing generalization ability.

### Unsupervised image classification

We evaluate our approach on unsupervised image classification for CIFAR-10 and CIFAR-100. Following established protocols, we report standard clustering metrics: Normalized Mutual Information (NMI) [46], Adjusted Normalized Mutual Information (AMI) [47], and Adjusted Rand-Index (ARI) [48]. We compare our approach with strong baselines including reproductions of MoCo-v2 [29], SimCLR [30], and the recent CueCo method [7]. All methods were evaluated using consistent hyperparameter settings for fair comparison.

Quantitative results are presented in Table 5. Our method consistently outperforms comparison methods across all metrics on both CIFAR-10 and CIFAR-100, indicating substantial improvement in clustering quality. On CIFAR-10, our method achieves state-of-the-art performance with an NMI of **86.59%**, AMI of **86.60%**, and ARI of **85.89%**. This represents a remarkable leap compared to the previous best-performing baseline, CueCo, improving ARI by over **32%** (85.89% vs. 53.87%). This margin indicates that our approach generates highly coherent clusters aligning closely with true semantic classes, minimizing confusion between categories. On the more challenging CIFAR-100, containing significantly more fine-grained categories, the superiority of our method is even more pronounced. We achieve an NMI of **60.15%** and ARI of **45.61%**. In contrast, the strongest baseline (CueCo) only reaches an ARI of 11.35%. Our method effectively **quadruples** the ARI score compared to baselines. This highlights the robustness of our framework in handling complex distributions with many classes, proving its ability to disentangle fine-grained semantic features where traditional methods often struggle.

**Table 5:** Unsupervised image classification results on CIFAR-10 and CIFAR-100 datasets. We report Normalized Mutual Information (NMI), Adjusted Mutual Information (AMI), and Adjusted Rand Index (ARI).

Method	CIFAR-10			CIFAR-100		
	NMI	AMI	ARI	NMI	AMI	ARI
MoCo-v2 [29]	60.96	60.88	28.95	51.77	45.84	9.95
SimCLR [30]	69.03	68.98	53.14	50.75	44.60	11.15
CueCo [7]	69.33	69.01	53.87	52.37	46.31	11.35
**OUR**	**86.59**	**86.60**	**85.89**	**60.15**	**56.15**	**45.61**

### Computational efficiency comparison

To further evaluate the practical utility of PEC-CDC, we compare its training efficiency with representative baseline methods under the same ResNet-18 backbone on CIFAR-10. All methods are evaluated using an input size of 32×32, a batch size of 256, and an NVIDIA GeForce RTX 2080 Ti GPU. In addition to clustering and classification performance, we report the number of parameters, backbone FLOPs, average training step time, and peak GPU memory consumption.

As shown in Table 6, PEC-CDC contains 21.81M parameters, which is higher than several contrastive baselines and comparable to methods with additional projection or prediction heads, such as SimSiam, VICReg, and Barlow Twins. This increase mainly comes from the additional PEC module, including the frozen teacher and cluster-specific student networks. However, a larger parameter count does not necessarily imply a longer training time. In PEC-CDC, the same ResNet-18 backbone architecture is used across all methods, as reflected by the identical backbone FLOPs. The additional PEC components operate on latent representations rather than full-resolution inputs. Moreover, the teacher network is frozen and does not require gradient updates, and the PEC optimization applies the stop-gradient operation to prevent the regression loss from backpropagating through the encoder. During PEC optimization, each selected sample updates only the student associated with its assigned cluster. Therefore, the extra parameters introduced by the PEC module mainly increase calibration-side storage and introduce only limited additional optimization cost.

**Table 6:** Computational efficiency comparison on CIFAR-10 using a ResNet-18 backbone.

Method	Params	Backbone FLOPs	Avg. step	Peak GPU mem.
	(M)	(G)	(ms)	(MB)
BYOL	16.44	0.0744	347.1	3493.1
DINO	17.99	0.0744	366.4	3523.5
SimSiam	22.72	0.0744	291.5	3186.8
MoCo-v2	**12.75**	0.0744	402.2	3490.8
MoCo-v3	16.43	0.0744	405.3	3488.5
ReSSL	14.32	0.0744	298.5	3511.8
VICReg	20.63	0.0744	308.3	3192.3
SwAV	12.87	0.0744	299.3	3074.9
SimCLR	**12.75**	0.0744	303.6	3061.0
Barlow Twins	20.63	0.0744	317.8	3201.6
PEC-CDC (Ours)	21.81	0.0744	**149.9**	**2310.0**

PEC-CDC achieves the lowest average training step time of 149.9 ms and the lowest peak GPU memory consumption of 2310.0 MB among the compared methods under the same experimental setting. These results are consistent with the theoretical complexity analysis, which shows that the main additional PEC-related computation for confidence estimation is linear in the number of samples and clusters and does not involve quadratic pairwise comparisons or global graph construction. Therefore, PEC-CDC improves clustering reliability through prediction error-based calibration while maintaining favorable computational efficiency and practical scalability.

### Robustness evaluation under noisy pseudo-labels

To further evaluate the robustness of PEC-CDC under challenging self-training conditions, we conducted an additional experiment on CIFAR-10 by injecting random noise into the pseudo-labels during training. This experiment was designed to examine whether the proposed prediction error-based calibration mechanism can maintain reliable clustering performance when pseudo-labels are partially corrupted. Specifically, we randomly replaced a fixed proportion of pseudo-labels with incorrect class assignments and compared PEC-CDC with the baseline CDC under the same noise ratios. All experiments were conducted for 10 epochs using the same random seed. The results are reported in Table 7.

**Table 7:** Robustness comparison between PEC-CDC and CDC in terms of clustering accuracy (ACC) under different pseudo-label noise ratios on CIFAR-10.

Noise ratio	0%	10%	20%	30%	40%
PEC-CDC	92.73%	92.26%	92.15%	91.32%	91.06%
CDC	90.25%	89.65%	89.93%	89.76%	89.17%

As shown in Table 7, PEC-CDC consistently outperforms CDC under all pseudo-label noise ratios. Without injected noise, PEC-CDC achieves an accuracy of 92.73%, compared with 90.25% for CDC. When the noise ratio increases to 40%, PEC-CDC still maintains an accuracy of 91.06%, whereas CDC reaches 89.17%. Across all noise levels, PEC-CDC preserves a performance advantage of approximately 1.56 to 2.61 percentage points over CDC. These results indicate that the proposed prediction error-based calibration mechanism remains effective when pseudo-labels are partially corrupted. Compared with CDC, which relies on calibrated softmax probabilities, PEC-CDC evaluates pseudo-label reliability through sample–cluster distributional consistency. This helps reduce the negative influence of unreliable pseudo-labels during self-training, allowing PEC-CDC to maintain consistently higher clustering performance under noisy pseudo-label conditions.

### Ablation Study

To verify the effectiveness of the proposed PEC-CDC framework and investigate the optimal integration strategy of the Prediction Error-based Classification (PEC) module, we conducted an ablation study on CIFAR-10. We designed three distinct configurations to analyze PEC module function when replacing different components of the original CDC architecture. Variants are defined as follows:

- **CDC (Baseline) [15]:** The original Calibrated Deep Clustering method, employing a standard dual-head structure consisting of a Clustering Head and a Calibration Head, both operating on softmax probabilities.
- **PEC-Clu-CDC:** A variant investigating the classification capability of the PEC module. Here, we *retain* the original Calibration Head of CDC but *replace* the Clustering Head with the PEC module. The PEC module is directly responsible for determining class assignments based on regression errors, while the probability-based calibration head remains auxiliary.
- **PEC-CDC (Ours):** The proposed framework, where we *retain* the Clustering Head of CDC but *replace* the Calibration Head with the PEC module. This design utilizes PEC regression error as a robust uncertainty metric to calibrate semantic representations learned by the Clustering Head.

Quantitative results are summarized in Table 8. Replacing the standard Clustering Head with the PEC module yields noticeable performance gain, with Top-1 accuracy rising from 89.09% to 90.76% and ARI improving by over 3% (79.23% → 82.41%). This indicates that the regression-based classification mechanism of PEC is inherently more discriminative than the standard softmax-based clustering head used in CDC, confirming the robustness of prediction error as a classification signal. Crucially, our proposed **PEC-CDC** framework significantly outperforms the **PEC-Clu-CDC** variant. Top-1 accuracy increases from 90.76% to 92.71%, and ARI sees an additional boost of roughly 3% (82.41% → 85.44%). This comparison reveals a key architectural insight: while PEC is a strong classifier on its own, its most effective role within this framework is as a *calibrator*. By assigning the classification task back to the Clustering Head (which excels at semantic separation when properly guided) and using the PEC module solely to provide accurate, error-based confidence estimation (replacing the weaker probability-based Calibration Head), we achieve the best synergy. This demonstrates that the specific configuration of PEC-CDC—using prediction error to calibrate semantic clustering—is the optimal design choice.

**Table 8:** Ablation study on CIFAR-10. We evaluate the impact of replacing either the Clustering Head (PEC-Clu-CDC) or the Calibration Head (PEC-CDC) with the PEC module compared to the CDC baseline.

Method	Classification		Clustering Metrics		
	Top-1	Top-5	NMI	AMI	ARI
CDC [15]	89.09	98.89	82.27	82.26	79.23
PEC-Clu-CDC	90.76	99.63	84.73	84.72	82.41
**PEC-CDC (Ours)**	**92.71**	**99.79**	**86.60**	**86.60**	**85.44**

### Visualization of representation evolution

To provide a qualitative assessment of the PEC-CDC framework, we visualize the evolution of feature distributions and classification behaviors throughout the training process. Specifically, we employ t-SNE [49] to project high-dimensional latent features into a 2D space and utilize Confusion Matrices to analyze class-wise prediction consistency. We compare the states of two internal components of the proposed PEC-CDC framework, namely the retained Clustering Head and the PEC module that replaces the original Calibration Head of CDC, at two distinct stages: initialization before training and convergence after training.

**Fig 2:**
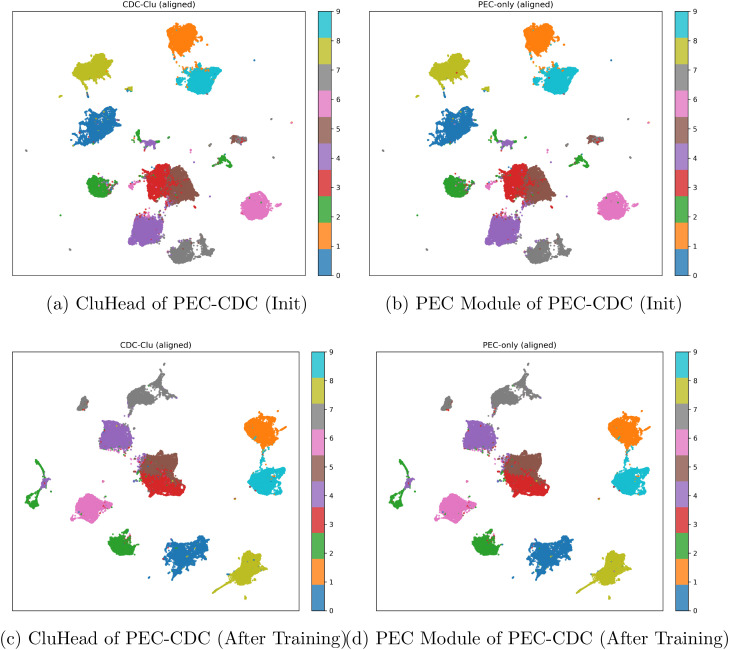
t-SNE Visualization of Feature Evolution. (a) and (b) show the initial feature distributions produced by the retained Clustering Head and the PEC module in PEC-CDC, respectively, where classes are indistinguishable. (c) and (d) show their corresponding feature distributions after training with PEC-CDC, demonstrating clear semantic separation and compact clustering.

Fig 2 illustrates t-SNE projections of learned representations at different training stages. At the beginning of training (Fig 2a and 2b), both the retained Clustering Head and the PEC module that replaces the original Calibration Head of CDC exhibit significantly overlapped feature spaces with scattered representations and no distinct semantic boundaries.

This lack of discernible cluster structure reflects random initialization of network parameters, indicating the model has not yet captured class-specific discriminative features. In contrast, after training with the proposed PEC-CDC framework (Fig 2c and 2d), a dramatic transformation is observed. Features learned by the retained Clustering Head form highly compact and well-separated clusters with clear inter-class margins, while the PEC module demonstrates a structured manifold where student networks successfully regress to the teacher target space within distinct regions.

To further support the qualitative observations in Fig 2, we report two quantitative cluster quality metrics, namely the Silhouette Score and the Davies–Bouldin Index. A higher Silhouette Score indicates better intra-cluster compactness and inter-cluster separation, whereas a lower Davies–Bouldin Index indicates better cluster quality. The results are summarized in Table 9.

**Table 9:** Quantitative cluster quality metrics corresponding to Fig 2.

Stage	Component	Silhouette Score	Davies–Bouldin Index
Initialization	CluHead of PEC-CDC	0.0371	3.6785
Initialization	PEC Module of PEC-CDC	0.0363	3.6829
After training	CluHead of PEC-CDC	0.0754	2.7034
After training	PEC Module of PEC-CDC	0.0737	2.7042

As shown in Table 9, both internal components exhibit consistent quantitative improvement after training. For the retained Clustering Head, the Silhouette Score increases from 0.0371 to 0.0754, while the Davies–Bouldin Index decreases from 3.6785 to 2.7034. For the PEC module, the Silhouette Score increases from 0.0363 to 0.0737, and the Davies–Bouldin Index decreases from 3.6829 to 2.7042. These results are consistent with the visual trend in Fig 2, showing that PEC-CDC improves intra-cluster compactness and inter-cluster separability during training. Moreover, the close metric values between the retained Clustering Head and the PEC module after training suggest that the two components achieve comparable cluster quality. Therefore, the combined visual and quantitative evidence supports the effectiveness of the proposed hybrid optimization strategy in organizing representations and improving cluster quality.

**Fig 3:**
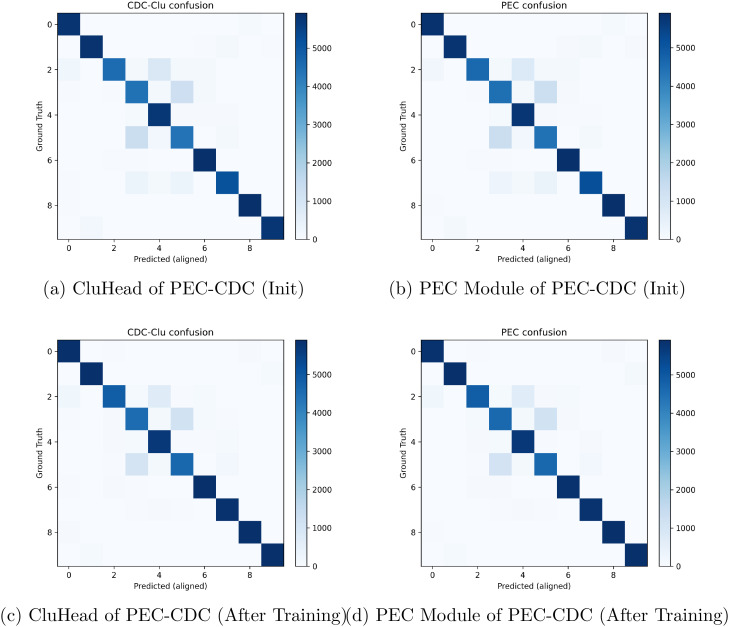
Evolution of Classification Consistency. (a) and (b) depict the confusion matrices of the retained Clustering Head and the PEC module in PEC-CDC at initialization, showing random predictions. (c) and (d) illustrate their corresponding results after training, where the pronounced diagonal indicates high classification accuracy and successful alignment between these two internal components of PEC-CDC.

Fig 3 displays confusion matrices, providing a granular view of prediction accuracy and consistency across training stages. Before training (Fig 3a and 3b), both the retained Clustering Head and the PEC module in PEC-CDC exhibit a dispersed distribution of predictions. The absence of a dominant diagonal indicates that initial predictions are virtually random, characterized by high confusion rates between disparate semantic classes.

Upon completion of training (Fig 3c and 3d), both the retained Clustering Head and the PEC module demonstrate strong diagonal dominance. Distinct dark blue diagonal elements signify high true-positive rates, while off-diagonal elements are effectively suppressed to near zero. This confirms that the PEC-CDC framework not only aligns latent features but also establishes accurate decision boundaries. This ensures that both the PEC module, which replaces the original Calibration Head of CDC and serves as a regression-based confidence estimator, and the retained Clustering Head, which serves as the semantic classifier, converge to a consistent and accurate state, validating the effectiveness of our hybrid optimization strategy.

### Analysis of prediction error distribution

**Fig 4:**
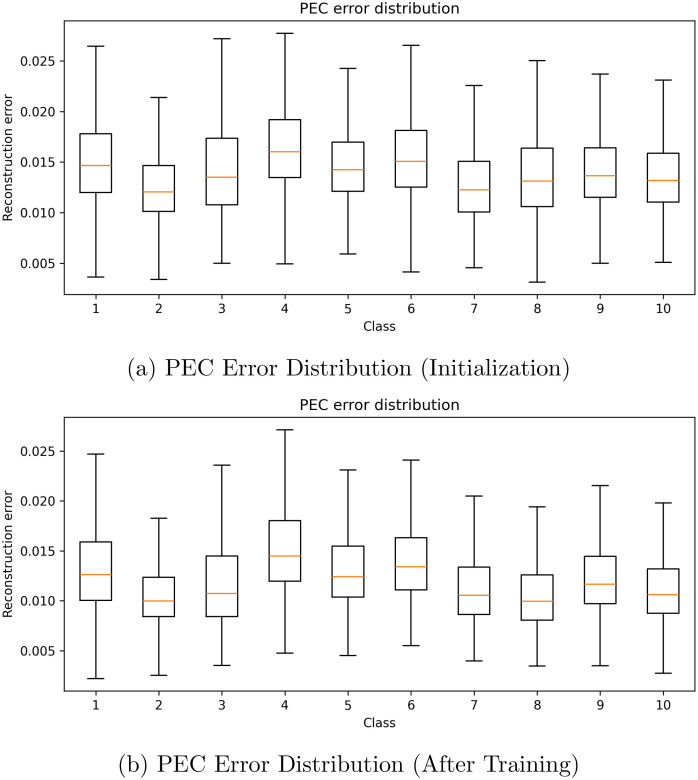
Boxplot of PEC Reconstruction Errors. The figure compares the reconstruction error distributions of the PEC module in PEC-CDC across ten classes before and after training. The significant reduction and stabilization of errors support the effectiveness of the PEC module in learning cluster-specific manifolds.

To further investigate the reliability of the PEC module as a confidence estimator, we analyze the distribution of reconstruction errors across different classes using boxplots. Fig 4 compares PEC error profiles at initialization and after convergence of the PEC-CDC framework. As illustrated in Fig 4a (Initialization), reconstruction errors for all classes are characterized by relatively high median values and large variances. This initial state reflects the fact that student networks have not yet learned specific data manifolds defined by the random teacher network. High overlap in error ranges across different classes indicates a lack of discriminative confidence, which would lead to unreliable pseudo-label selection in traditional self-training setups. In contrast, Fig 4b shows error distribution after training. We observe a significant downward shift in median reconstruction error for all categories, with interquartile ranges (IQRs) becoming much more compact. This compression of the error profile demonstrates that each class-specific student network has successfully specialized in the feature distribution of its corresponding cluster. Consistently low and stable error levels across ten classes confirm that the PEC module provides a reliable and absolute metric for distributional support. This high degree of regression consistency ensures that only samples with high structural certainty are assigned high confidence scores, effectively filtering out noisy samples and preventing accumulation of errors during self-training.

## Conclusion

In this paper, we presented PEC-CDC, a novel deep clustering framework designed to address the critical issue of overconfidence in unsupervised self-training. While traditional methods and the original CDC framework rely on relative softmax-derived probabilities—which are inherently limited by inter-class normalization—our approach shifts the focus toward absolute distributional consistency. By strategically replacing the probability-based calibration head with a regression-based PEC head, we established a more reliable uncertainty metric based on frozen Teacher-Student consistency.

Our experimental results, both qualitative and quantitative, underscore the efficacy of this architectural evolution. The PEC module effectively filters out noisy pseudo-labels by quantifying the absolute alignment between samples and cluster manifolds, leading to a more robust and semantically coherent optimization process. Notably, PEC-CDC achieved state-of-the-art performance across multiple benchmarks, including a four-fold increase in the Adjusted Rand Index (ARI) on the challenging CIFAR-100 dataset compared to previous baselines.

Ultimately, this research demonstrates that absolute prediction error is a superior indicator for confidence assessment in unsupervised settings. By mitigating the root cause of overconfidence, our work provides a robust foundation for developing trustworthy and autonomous machine learning systems. Future research will explore the integration of this regression-based calibration with broader backbone architectures and its extension to more complex, cross-modal data discovery tasks.

## References

[1] Rezaei S, Chegeni A, Javadpour A, VafaeiSadr A, Cao L, Röttgering H, et al. Bridging gaps with computer vision: AI in (bio)medical imaging and astronomy. Astronomy and Computing. 2025;51:100921. doi: 10.1016/j.ascom.2024.100921

[2] Arefin MR, Zhang Y, Baratin A, Locatello F, Rish I, Liu D. Unsupervised concept discovery mitigates spurious correlations. In: Proceedings of the 41st International Conference on Machine Learning, 2024. 1672–88.

[3] Obikane S, Tagawa H, Aoki Y. Unsupervised Metric Learning for Expressing Color and Shape Information to Uncover Abstract Connections within Image Datasets. In: Pattern Recognition; 2025. p. 15–30.

[4] Lu Y, Li H, Li Y, Lin Y, Peng X. A survey on deep clustering: from the prior perspective. Vicinagearth. 2024;1(1). doi: 10.1007/s44336-024-00001-w

[5] Xie J, Girshick R, Farhadi A. Unsupervised deep embedding for clustering analysis. In: Proceedings of The 33rd International Conference on Machine Learning, 2016. 478–87.

[6] Van Gansbeke W, Vandenhende S, Georgoulis S, Proesmans M, Van Gool L. SCAN: Learning to Classify Images Without Labels. In: European Conference on Computer Vision, 2020. 268–85.

[7] Giakoumoglou N, Stathaki T. Cluster Contrast for Unsupervised Visual Representation Learning. In: 2025 IEEE International Conference on Image Processing (ICIP), 2025. 133–8. 10.1109/icip55913.2025.11084524

[8] Caron M, Bojanowski P, Joulin A, Douze M. Deep Clustering for Unsupervised Learning of Visual Features. Lecture Notes in Computer Science. Springer International Publishing. 2018. p. 139–56. 10.1007/978-3-030-01264-9_9

[9] Caron M, Misra I, Mairal J, Goyal P, Bojanowski P, Joulin A. Unsupervised learning of visual features by contrasting cluster assignments. In: Proceedings of the 34th International Conference on Neural Information Processing Systems, 2020. 9912–24.

[10] Amini M-R, Feofanov V, Pauletto L, Hadjadj L, Devijver É, Maximov Y. Self-training: A survey. Neurocomputing. 2025;616:128904. doi: 10.1016/j.neucom.2024.128904

[11] Zhang S, Yan C, Yang J, Ren C, Bai J, Li T, et al. RoNID: New Intent Discovery with Generated-Reliable Labels and Cluster-friendly Representations. Lecture Notes in Computer Science. Springer Nature Singapore. 2024. p. 104–19. 10.1007/978-981-97-5569-1_7

[12] Li D, Zhang J, Wu K, Shi Y, Han Y. Pseudo-label refinement via hierarchical contrastive learning for source-free unsupervised domain adaptation. Pattern Recognition Letters. 2024;186:236–42. doi: 10.1016/j.patrec.2024.10.006

[13] Sun H, Gao Y, Ma S. Pseudo labels purification for unsupervised person re-identification. Signal, Image and Video Processing. 2025;19(1):24.

[14] Rai S, Thakur RS, Jangid K, Kurmi VK. Label Calibration in Source Free Domain Adaptation. In: 2025 IEEE/CVF Winter Conference on Applications of Computer Vision (WACV), 2025. 6446–55. 10.1109/wacv61041.2025.00628

[15] Jia Y, Cheng J, L I U H, Hou J. Towards calibrated deep clustering network. In: 2025.

[16] Murugesan B, Adiga Vasudeva S, Liu B, Lombaert H, Ben Ayed I, Dolz J. Neighbor-aware calibration of segmentation networks with penalty-based constraints. Med Image Anal. 2025;101:103501. doi: 10.1016/j.media.2025.103501 39978014

[17] Zhang S, Xie L. Advancing neural network calibration: The role of gradient decay in large-margin Softmax optimization. Neural Netw. 2024;178:106457. doi: 10.1016/j.neunet.2024.106457 38908166

[18] Niu C, Shan H, Wang G. SPICE: Semantic Pseudo-Labeling for Image Clustering. IEEE Trans Image Process. 2022;31:7264–78. doi: 10.1109/TIP.2022.3221290 36378790 PMC9767807

[19] Wang Z, Loog M. Enhancing Classifier Conservativeness and Robustness by Polynomiality. In: 2022 IEEE/CVF Conference on Computer Vision and Pattern Recognition (CVPR), 2022. 13317–26. 10.1109/cvpr52688.2022.01297

[20] Zajac M, Tuytelaars T, van de Ven GM. Prediction error-based classification for class-incremental learning. In: 2024.

[21] Chang L, Niu X, Li Z, Zhang Z, Li S, Fournier-Viger P. ULDC: uncertainty-based learning for deep clustering. Appl Intell. 2024;55(3). doi: 10.1007/s10489-024-06125-2

[22] Yang Q, Li Z, Han G, Gao W, Zhu S, Wu X, et al. An improvement of spectral clustering algorithm based on fast diffusion search for natural neighbor and affinity propagation. J Supercomput. 2022;78(12):14597–625. doi: 10.1007/s11227-022-04456-w

[23] Yang Q-F, Gao W-Y, Han G, Li Z-Y, Tian M, Zhu S-H, et al. HCDC: A novel hierarchical clustering algorithm based on density-distance cores for data sets with varying density. Information Systems. 2023;114:102159. doi: 10.1016/j.is.2022.102159

[24] Yang Q, Han G, Gao W, Yang Z, Zhu S, Deng Y. A Robust Learning Membership Scaling Fuzzy C-Means Algorithm Based on New Belief Peak. IEEE Trans Fuzzy Syst. 2023;31(12):4486–500. doi: 10.1109/TFUZZ.2023.1234567

[25] Hu Z, Xu X, Su Q, Zhu H, Guo J. Grey prediction evolution algorithm for global optimization. Applied Mathematical Modelling. 2020;79:145–60. doi: 10.1016/j.apm.2019.10.026

[26] Zhang R, Mao S, Kang Y. A novel traffic flow prediction model: Variable order fractional grey model based on an improved grey evolution algorithm. Expert Systems with Applications. 2023;224:119943. doi: 10.1016/j.eswa.2023.119943

[27] Tong W, Liu D, Hu Z, Su Q. Hybridizing genetic algorithm with grey prediction evolution algorithm for solving unit commitment problem. Appl Intell. 2023;53(17):19922–39. doi: 10.1007/s10489-023-04527-2

[28] Shaheen A, Mrabah N, Ksantini R, Alqaddoumi A. Rethinking deep clustering paradigms: Self-supervision is all you need. Neural Netw. 2025;181:106773. doi: 10.1016/j.neunet.2024.106773 39383676

[29] Chen X, Fan H, Girshick R, He K. Improved Baselines with Momentum Contrastive Learning. In: 2020. https://doi.org/arXiv:2003.04297

[30] Chen T, Kornblith S, Norouzi M, Hinton G. In: Proceedings of the 37th International Conference on Machine Learning, 2020. 1597–607.

[31] Chen X, Xie S, He K. An Empirical Study of Training Self-Supervised Vision Transformers. In: 2021 IEEE/CVF International Conference on Computer Vision (ICCV), 2021. 9620–9. 10.1109/iccv48922.2021.00950

[32] Caron M, Touvron H, Misra I, Jegou H, Mairal J, Bojanowski P, et al. Emerging Properties in Self-Supervised Vision Transformers. In: 2021 IEEE/CVF International Conference on Computer Vision (ICCV), 2021. 9630–40. 10.1109/iccv48922.2021.00951

[33] Grill JB, Strub F, Altché F, Tallec C, Richemond P, Buchatskaya E, et al. Bootstrap your own latent - a new approach to self-supervised learning. In: Advances in Neural Information Processing Systems, 2020.

[34] Chen X, He K. In: Proceedings of the IEEE/CVF Conference on Computer Vision and Pattern Recognition (CVPR), 2021. 15750–8.

[35] Zbontar J, Jing L, Misra I, LeCun Y, Deny S. Barlow Twins: Self-Supervised Learning via Redundancy Reduction. In: Proceedings of the 38th International Conference on Machine Learning, 2021. 12310–20.

[36] Bardes A, Ponce J, LeCun Y. VICReg: Variance-Invariance-Covariance Regularization for Self-Supervised Learning. In: 2022.

[37] Zhu P, Li J, Wang Y, Xiao B, Zhang J, Lin W, et al. Boosting Pseudo-Labeling With Curriculum Self-Reflection for Attributed Graph Clustering. IEEE Trans Neural Netw Learn Syst. 2025;36(5):8346–59. doi: 10.1109/TNNLS.2024.3416167 39250368

[38] Guo C, Pleiss G, Sun Y, Weinberger KQ. On calibration of modern neural networks. In: Proceedings of the 34th International Conference on Machine Learning - Volume 70, 2017. 1321–30.

[39] Lakshminarayanan B, Pritzel A, Blundell C. Simple and scalable predictive uncertainty estimation using deep ensembles. In: Proceedings of the 31st International Conference on Neural Information Processing Systems, 2017. 6405–16.

[40] Wei H, Xie R, Cheng H, Feng L, An B, Li Y. Mitigating Neural Network Overconfidence with Logit Normalization. In: Proceedings of the 39th International Conference on Machine Learning, 2022. 23631–44.

[41] Xiao Y, Tang C, Zheng X, Yan W, Liu Y, Liu X. Mutual Calibration Network for Multi-View Clustering. IEEE Trans Circuits Syst Video Technol. 2026;36(1):393–405. doi: 10.1109/tcsvt.2025.3588889

[42] Krizhevsky A, Hinton G. Learning multiple layers of features from tiny images. Department of Computer Science, University of Toronto. 2009.

[43] Deng J, Dong W, Socher R, Li L-J, Kai Li, Li Fei-Fei. ImageNet: A large-scale hierarchical image database. In: 2009 IEEE Conference on Computer Vision and Pattern Recognition, 2009. 248–55. 10.1109/cvpr.2009.5206848

[44] Helber P, Bischke B, Dengel A, Borth D. EuroSAT: A Novel Dataset and Deep Learning Benchmark for Land Use and Land Cover Classification. IEEE J Sel Top Appl Earth Observations Remote Sensing. 2019;12(7):2217–26. doi: 10.1109/jstars.2019.2918242

[45] Zheng M, You S, Wang F, Qian C, Zhang C, Wang X. ReSSL: relational self-supervised learning with weak augmentation. In: Proceedings of the 35th International Conference on Neural Information Processing Systems, 2021. 2543–55.

[46] Li T, Ding C. The Relationships Among Various Nonnegative Matrix Factorization Methods for Clustering. In: Sixth International Conference on Data Mining (ICDM’06), 2006. 362–71. 10.1109/icdm.2006.160

[47] Vinh NX, Epps J, Bailey J. In: 2009. 1073–80.

[48] Hubert L, Arabie P. Comparing partitions. Journal of Classification. 1985;2(1):193–218. doi: 10.1007/bf01908075

[49] Maaten L v d, Hinton G. Visualizing data using t-SNE. Journal of Machine Learning Research. 2008;9(Nov):2579–605.

