# Supplementary material for: PEC-CDC: A prediction error-based calibration framework for robust unsupervised deep clustering
Source: PLoS One. 2026 Jun 25;21(6):e0351959. doi: 10.1371/journal.pone.0351959 (PMC13298931; doi:10.1371/journal.pone.0351959)
Supplement: Attachment — Submitted filename: Response to Reviewers.docx [file pone.0351959.s001.docx]

**Our response to the review comments**

First of all, we would like to thank the anonymous reviewers for their constructive comments to improve our manuscript (Manuscript ID: PONE-D-26-02968). We really appreciate their valuable time and great efforts in reviewing the manuscript. Please find below our detailed response to the comments point by point.

**Editor：1**

**Comment 1.1.** Figures 2, 3, and 4 need to be repositioned to appear directly after their first citation in the text. The current placement is misleading and disrupts the reading flow. For instance, Fig. 2 should be moved to Page 16, where the section "Visualization of Representation Evolution" begins.

**Response to Comment 1.1:** We thank the editor for the helpful comment. We have carefully revised the placement of Figs. 2, 3, and 4 to ensure that each figure appears directly after its first citation in the manuscript. Specifically, Fig. 2-4 have also been repositioned after the corresponding paragraphs in which they are first cited. **Please refer to Fig. 2 on Page 22, Fig. 3 on Page 23, and Fig. 4 on Page 24 of the revised manuscript.**

**Comment 1.2.** There is an inconsistency in model naming conventions between the tables and figures. Table 4 uses the identifiers "CD," "PEC-Clu-CDC," and "PEC-CDC," whereas Fig. 2 employs "CDC-Clu" and "PEC." A unified naming scheme should be adopted throughout the manuscript to avoid confusion.

**Response to Comment 1.2:** We thank the editor for pointing out this inconsistency. We have carefully checked the manuscript and unified the naming conventions across the tables, figures, captions, and corresponding textual descriptions. Specifically, the baseline method is now consistently referred to as “CDC,” the ablation variant is referred to as “PEC-Clu-CDC,” and the proposed method is referred to as “PEC-CDC.” In addition, the labels in the visualization figures have been revised to clearly indicate the internal components of PEC-CDC, namely “CluHead of PEC-CDC” and “PEC Module of PEC-CDC,” rather than using the ambiguous terms “CDC-Clu” and “PEC.” These revisions make the terminology consistent and reduce potential confusion for readers. **Please refer to Table 9, Figs. 2-4, and the corresponding descriptive paragraphs in the “Visualization of Representation Evolution” section on Pages 21-23 of the revised manuscript, marked in blue, for details.**

**Comment 1.3.** In Fig. 2, the visualization of feature evolution does not reveal sufficiently obvious or interpretable differences between the compared variants. The authors should either provide a more detailed discussion highlighting the specific distinctions, or consider supplementing the figure with quantitative metrics (e.g., silhouette score, Davies–Bouldin index) to support the visual claims.

**Response to Comment 1.3:** We thank the editor for the constructive suggestion. We have revised the discussion of Fig. 2 to make the interpretation of feature evolution clearer and more quantitative. Specifically, we added two cluster quality metrics, Silhouette Score and Davies-Bouldin Index, to supplement the t-SNE visualization. The added results show that, after training, both the retained Clustering Head and the PEC module achieve higher Silhouette Scores and lower Davies--Bouldin Index values, indicating improved intra-cluster compactness and inter-cluster separability, as shown in **Table 9 on Page 21**. We also expanded the corresponding analysis to better explain the differences between the initialization and representations after training. These revisions provide quantitative support for the visual claims in Fig. 2. The specific revisions are shown below:

**(Please refer to Table 9 and the corresponding paragraph on Page 21 of the revised manuscript, marked in blue, for details.)**

To further support the qualitative observations in Fig 2, we report two quantitative cluster quality metrics, namely the Silhouette Score and the Davies--Bouldin Index. A higher Silhouette Score indicates better intra-cluster compactness and inter-cluster separation, whereas a lower Davies--Bouldin Index indicates better cluster quality. The results are summarized in Table 9.

As shown in Table9, both internal components exhibit consistent quantitative improvement after training. For the retained Clustering Head, the Silhouette Score increases from 0.0371 to 0.0754, while the Davies--Bouldin Index decreases from 3.6785 to 2.7034. For the PEC module, the Silhouette Score increases from 0.0363 to 0.0737, and the Davies--Bouldin Index decreases from 3.6829 to 2.7042. These results are consistent with the visual trend in Fig 2, showing that PEC-CDC improves intra-cluster compactness and inter-cluster separability during training. Moreover, the close metric values between the retained Clustering Head and the PEC module after training suggest that the two components achieve comparable cluster quality. Therefore, the combined visual and quantitative evidence supports the effectiveness of the proposed hybrid optimization strategy in organizing representations and improving cluster quality.

**Comment 1.4.** Table 2 provides a performance comparison across various frameworks in terms of clustering accuracy. However, this represents only one dimension of evaluation. A comparison of training resources and computational efficiency (e.g., training time, GPU memory consumption, FLOPs, or number of parameters) under the same backbone network is also necessary to provide a complete assessment of practical utility.

**Response to Comment 1.4:** We thank the editor for the helpful suggestion. We agree that clustering accuracy alone is not sufficient to fully evaluate the practical utility of the proposed method. Accordingly, we added a new subsection entitled “Computational Efficiency Comparison” to provide a more comprehensive evaluation under the same ResNet-18 backbone. In this subsection, we compare the number of parameters, backbone FLOPs, average training step time, and peak GPU memory consumption across different methods. The added results show that although PEC-CDC introduces additional parameters due to the PEC module, it maintains favorable computational efficiency, achieving a lower average training step time and lower peak GPU memory consumption under the same experimental setting. We also added a short analysis to explain that the additional PEC components operate on latent representations, use a frozen teacher, and do not involve quadratic pairwise comparisons or global graph construction. The specific revisions are shown below:

**(Please refer to Table 6 and the corresponding paragraph in the “Computational Efficiency Comparison” subsection on Page 18-19 of the revised manuscript, marked in blue.)**

**Computational Efficiency Comparison**

To further evaluate the practical utility of PEC-CDC, we compare its training efficiency with representative baseline methods under the same ResNet-18 backbone on CIFAR-10. All methods are evaluated using an input size of 32 $\times$32, a batch size of 256, and an NVIDIA GeForce RTX 2080 Ti GPU. In addition to clustering and classification performance, we report the number of parameters, backbone FLOPs, average training step time, and peak GPU memory consumption.

As shown in Table 6, PEC-CDC contains 21.81M parameters, which is higher than several contrastive baselines and comparable to methods with additional projection or prediction heads, such as SimSiam, VICReg, and Barlow Twins. This increase mainly comes from the additional PEC module, including the frozen teacher and cluster-specific student networks. However, a larger parameter count does not necessarily imply a longer training time. In PEC-CDC, the same ResNet-18 backbone architecture is used across all methods, as reflected by the identical backbone FLOPs. The additional PEC components operate on latent representations rather than full-resolution inputs. Moreover, the teacher network is frozen and does not require gradient updates, and the PEC optimization applies the stop-gradient operation to prevent the regression loss from backpropagating through the encoder. During PEC optimization, each selected sample updates only the student associated with its assigned cluster. Therefore, the extra parameters introduced by the PEC module mainly increase calibration-side storage and introduce only limited additional optimization cost.

PEC-CDC achieves the lowest average training step time of 149.9 ms and the lowest peak GPU memory consumption of 2310.0 MB among the compared methods under the same experimental setting. These results are consistent with the theoretical complexity analysis, which shows that the main additional PEC-related computation for confidence estimation is linear in the number of samples and clusters and does not involve quadratic pairwise comparisons or global graph construction. Therefore, PEC-CDC improves clustering reliability through prediction error-based calibration while maintaining favorable computational efficiency and practical scalability.

**Comment 1.5.** The method is validated on standard benchmark datasets, which establishes its reliability. However, the evaluation feels incomplete. As the authors themselves state in the manuscript: "PEC-CDC enables more trustworthy and accurate autonomous data discovery in high-dimensional domains such as biomedical and satellite imaging." I strongly recommend extending the experimental validation to these downstream domains (e.g., biomedical imaging and satellite imaging). Doing so would significantly strengthen the completeness and practical impact of the study.

**Response to Comment 1.5:** We thank the reviewer for the constructive suggestion. We agree that evaluation on downstream high-dimensional visual domains can further strengthen the practical impact of the proposed method. Accordingly, we added an additional satellite imaging evaluation on the EuroSAT dataset, which contains Sentinel-2 satellite images from 10 land use and land cover classes. The added experiment evaluates whether PEC-CDC can generalize beyond standard natural image benchmarks to remote sensing scenarios with different visual patterns and spatial structures. The results show that PEC-CDC achieves the best Top-1 accuracy of 94.67% and a Top-5 accuracy of 99.96% under the ResNet-18 backbone, outperforming or matching the compared representative methods. These results indicate that PEC-CDC can learn discriminative representations in satellite imaging scenarios and further demonstrate its practical applicability to downstream high-dimensional data discovery tasks. The specific revisions are shown below:

(**Please refer to Table 4 and the fourth and fifth paragraphs of the “Linear Evaluation” section on Page 17 of the revised manuscript, marked in blue.**)

To further examine the generalization ability of PEC-CDC beyond standard natural image benchmarks, we additionally evaluated the learned representations on the EuroSAT dataset. EuroSAT is constructed from Sentinel-2 satellite images and contains 27,000 labeled and geo-referenced images from 10 land use and land cover classes [44]. Compared with CIFAR and ImageNet-style datasets, EuroSAT represents a remote sensing scenario with different visual patterns and spatial structures, providing a useful benchmark for evaluating cross-domain representation quality. Since not all baselines provided available EuroSAT results under the same setting, we compared PEC-CDC with several representative methods selected from Table 4, including BYOL [33], DINO [32], SimSiam [34], MoCo-v2 [29], MoCo-v3 [31], and SimCLR [30]. The results are summarized in Table 4.

As shown in Table 4, PEC-CDC achieves the best Top-1 accuracy of 94.67% on EuroSAT, outperforming all compared baselines. Specifically, it improves Top-1 accuracy by 1.97 percentage points over DINO and by 2.49 percentage points over BYOL. PEC-CDC also obtains a Top-5 accuracy of 99.96%, matching the best result among the compared methods. These results indicate that the learned representations remain discriminative in the satellite imaging domain, where image characteristics differ from standard natural images. This further suggests that PEC-CDC has practical applicability beyond standard object-centered benchmarks.

**Reviewer: 1**

**Comment 1.1.** In Introduction section, while the motivation for replacing probability metrics with prediction error is compelling, the authors could further strengthen the motivation by explicitly defining the notion of robustness targeted and more clearly articulating the novelty of moving beyond probability-based calibration. The authors claim robustness in terms of: noise tolerance, avoiding local optima, stability under ambiguous boundaries. However, the word “robust” still appears more as an outcome than a defined objective. “Robust to what specific perturbations or conditions?”

**Response to Comment 1.1:** We sincerely thank the reviewer for this insightful comment. We agree that the notion of robustness should be defined more explicitly and that the novelty of moving beyond probability-based calibration should be more clearly articulated. To address this issue, we have revised the manuscript in two sections: the Introduction and the Robustness Evaluation in the Experimental Results and Analysis section.

In the Introduction, we have clarified that the robustness targeted in this study refers to the ability of the model to reduce the selection of unreliable pseudo-labels under noisy or uncertain self-training conditions, including noisy samples, early-stage prediction errors, and samples near ambiguous class boundaries. We also explained that probability-based calibration may still assign high confidence to uncertain samples because softmax confidence is derived from relative class probabilities. The specific revisions are shown below:

(**Please refer to the second and third paragraphs in the Introduction on Pages 2-3, marked in blue in the revised manuscript.**)

This limitation suggests that, to improve robustness during iterative self-training, confidence estimation should not only calibrate probability scores but also reduce the selection of unreliable pseudo-labels. During learning, noisy samples, early-stage misassignments, and samples near ambiguous class boundaries may still be assigned high-confidence pseudo-labels. Existing probability-based refinement methods, including calibrated deep clustering methods, derive confidence from relative class probabilities. Thus, even after calibration, a sample may be regarded as reliable simply because one class score is higher than the others, although its cluster membership remains uncertain. Such unreliable pseudo-labels may be repeatedly reused in subsequent optimization, amplifying errors and reducing training stability. A more robust calibration mechanism should therefore evaluate whether a sample conforms to the distributional structure of a specific cluster, rather than only whether it obtains a relatively high probability.

Once such overconfident but incorrect pseudo-labels are selected, their errors can be reinforced in later iterations, reducing optimization stability and increasing the risk of poor local optima.

In this context, robustness refers to the ability of the model to avoid selecting unreliable pseudo-labels under noisy or uncertain training conditions, including noisy samples, early-stage misassignments, and samples near ambiguous class boundaries.

In the Introduction, we further clarified that the novelty of PEC-CDC lies in replacing probability-based confidence with prediction error-based calibration. Instead of evaluating whether a sample obtains a relatively high probability, PEC-CDC evaluates whether the sample conforms to a cluster-specific distributional structure. This revision better explains why prediction error is more suitable for reducing unreliable pseudo-label selection during self-training. The specific revisions are shown below:

(**Please refer to the fourth and fifth paragraphs in the Introduction on Page 3, marked in blue in the revised manuscript.**)

This shift makes pseudo-label selection less sensitive to noisy samples, early-stage misassignments, and ambiguous decision boundaries, thereby improving the robustness of self-training.

Compared with probability-based confidence, prediction error evaluates whether a sample is well supported by a cluster-specific distribution rather than whether it merely obtains the largest relative probability. Therefore, samples affected by noise or located near ambiguous boundaries tend to produce larger errors and are less likely to be selected as reliable pseudo-labels.

To further support this motivation, we also added a new subsection entitled “Robustness Evaluation under Noisy Pseudo-labels” in the Experimental Results and Analysis section. In this subsection, we conduct a robustness experiment under noisy pseudo-label settings. Specifically, we injected different proportions of random noise into the pseudo-labels during training on CIFAR-10 and compared PEC-CDC with the baseline CDC under the same noise ratios. The results show that PEC-CDC consistently achieves higher clustering accuracy than CDC across all tested noise ratios. These results demonstrate that PEC-CDC can better maintain clustering performance when pseudo-labels are partially corrupted, confirming its robustness under noisy self-training conditions. The specific revisions are shown below:

(**Please refer to Table 7 and the Subsection “Robustness Evaluation under Noisy Pseudo-labels” on Pages 19-20 of the revised manuscript, marked in blue.**)

**Robustness Evaluation under Noisy Pseudo-labels**

To further evaluate the robustness of PEC-CDC under challenging self-training conditions, we conducted an additional experiment on CIFAR-10 by injecting random noise into the pseudo-labels during training. This experiment was designed to examine whether the proposed prediction error-based calibration mechanism can maintain reliable clustering performance when pseudo-labels are partially corrupted. Specifically, we randomly replaced a fixed proportion of pseudo-labels with incorrect class assignments and compared PEC-CDC with the baseline CDC under the same noise ratios. All experiments were conducted for 10 epochs using the same random seed. The results are reported in Table 7.

As shown in Table 7, PEC-CDC consistently outperforms CDC under all pseudo-label noise ratios. Without injected noise, PEC-CDC achieves an accuracy of 92.73%, compared with 90.25% for CDC. When the noise ratio increases to 40%, PEC-CDC still maintains an accuracy of 91.06%, whereas CDC reaches 89.17%. Across all noise levels, PEC-CDC preserves a performance advantage of approximately 1.56 to 2.61 percentage points over CDC. These results indicate that the proposed prediction error-based calibration mechanism remains effective when pseudo-labels are partially corrupted. Compared with CDC, which relies on calibrated softmax probabilities, PEC-CDC evaluates pseudo-label reliability through sample--cluster distributional consistency. This helps reduce the negative influence of unreliable pseudo-labels during self-training, allowing PEC-CDC to maintain consistently higher clustering performance under noisy pseudo-label conditions.

**Comment 1.2.** In Related Work Section, while the related work section provides a comprehensive overview of existing self-training and calibration-based deep clustering methods, it would benefit from a more explicit positioning of the proposed approach relative to prior work. In particular, the authors are encouraged to include a summarized comparison (a concise table) that contrasts the proposed PEC-CDC framework with representative prior methods in terms of calibration signal (prediction error vs. probability), reliance on softmax confidence, robustness to noisy pseudo-labels, and computational overhead. Such a comparison would help clearly establish the novelty and practical advantages of the proposed method over existing approaches

**Response to Comment 1.2:** We sincerely thank the reviewer for this constructive suggestion. We agree that a more explicit comparison with representative prior methods is important for clarifying the novelty and practical advantages of PEC-CDC. To address this issue, we have revised the Related Work section by adding a new subsection and a concise comparison table.
 In the revised manuscript, we added a new subsection titled “Advantages of PEC-CDC over Representative Prior Methods” to explicitly discuss how PEC-CDC differs from existing methods. Specifically, we compare PEC-CDC with contrastive self-supervised learning methods, non-contrastive self-supervised learning methods, clustering-oriented deep clustering methods, and calibrated deep clustering methods. This added discussion explains that most prior methods either improve representation quality indirectly or rely on prototype assignments, neighborhood consistency, or probability-based confidence for pseudo-label selection. In contrast, PEC-CDC uses prediction error to evaluate sample--cluster distributional consistency, which helps reduce the selection of unreliable pseudo-labels during self-training. The specific revisions are shown below:
(**Please refer to the newly added subsection in the Related Work section on Page 5 of the revised manuscript, marked in blue.**)

**Advantages of PEC-CDC over Representative Prior Methods**

To highlight the advantages of PEC-CDC over representative prior methods, Table 1 provides a summarized comparison in terms of calibration signal, reliance on softmax confidence, robustness to noisy pseudo-labels, and computational overhead. The comparison covers representative contrastive and non-contrastive self-supervised learning (SSL) methods, clustering-oriented deep clustering methods, calibrated deep clustering methods, and our proposed PEC-CDC framework.

As summarized in Table 1, the advantages of PEC-CDC mainly arise from its prediction error-based calibration mechanism. Contrastive and non-contrastive SSL methods primarily improve representation quality, but they do not explicitly calibrate pseudo-label reliability; therefore, their robustness to noisy pseudo-labels is only indirect. Clustering-oriented deep clustering methods rely on prototype assignments, neighborhood consistency, or prediction probabilities. When early cluster assignments are unreliable, incorrect pseudo-labels may be repeatedly reinforced during self-training.

We also added a concise comparison table to present the differences between PEC-CDC and representative prior methods more clearly. The table compares the methods in terms of calibration signal, reliance on softmax confidence, robustness to noisy pseudo-labels, and computational overhead, as suggested by the reviewer. This comparison shows that PEC-CDC differs from prior methods by using prediction error rather than softmax-derived probability as the calibration signal. It also illustrates that PEC-CDC reduces reliance on softmax confidence, improves robustness to noisy pseudo-labels, and maintains moderate computational overhead. The specific revisions are shown below:
(**Please refer to Table 1 in the Related Work section on Page 6 of the revised manuscript, marked in blue.**)

Calibrated deep clustering methods further improve pseudo-label selection through calibrated probabilities, but they still depend on softmax-derived confidence, which may assign high confidence to ambiguous samples. In contrast, PEC-CDC replaces probability-based calibration with prediction error-based calibration. Since prediction error measures the distributional consistency between a sample and cluster-specific regression models, ambiguous samples with weak distributional support tend to receive lower confidence and are less likely to be selected. Therefore, PEC-CDC improves robustness to noisy pseudo-labels while maintaining moderate computational overhead, as it does not require large-scale pairwise comparisons or global graph construction.

**Comment 1.3.** In Results and Discussion section, the experimental evaluation is conducted on CIFAR-10, CIFAR-100, and ImageNet-100, which are standard and appropriate benchmarks for unsupervised clustering. However, given the paper’s emphasis on robustness and calibration under noisy pseudo-labels, the current dataset selection primarily reflects clean, in-distribution settings. The authors are encouraged to include additional evaluations under challenging conditions such as noisy or corrupted data, out-of-distribution scenarios to more convincingly demonstrate the robustness and generalizability of the proposed PEC-CDC framework. The paper does not provide an explicit computational complexity analysis of the proposed PEC-CDC framework. Given that the method introduces an additional PEC Head and modifies the calibration mechanism within a self-training pipeline, it is important to clarify the time and space complexity relative to the baseline CDC and other deep clustering methods. The authors are encouraged to include a theoretical complexity analysis and/or empirical runtime comparisons to demonstrate the scalability and practical feasibility of the proposed approach. While the paper provides strong empirical motivation and cites prior work suggesting that prediction error correlates with uncertainty, it does not present any theoretical reasoning or formal justification linking prediction error to clustering reliability in the proposed framework. In particular, there is no analysis or assumption explaining why lower prediction error should consistently indicate higher sample–cluster consistency or more reliable pseudo-labels. Even an informal theoretical argument, simplified model, or assumption-based analysis would strengthen the generality and credibility of the proposed calibration mechanism, which currently appears largely empirical.

**Response to Comment 1.3:** We sincerely thank the reviewer for these insightful and constructive comments. We agree that the robustness, scalability, and theoretical basis of PEC-CDC should be further clarified to strengthen the credibility of the proposed framework. To address these concerns, we have revised the manuscript in four specific parts: Robustness Evaluation, Computational Efficiency Comparison, Computational Complexity Analysis, and the Theoretical Link Between Prediction Error and Clustering Reliability.

To evaluate PEC-CDC under more challenging self-training conditions, we added an experiment on CIFAR-10 with noisy pseudo-labels. Specifically, different proportions of pseudo-labels were randomly replaced with incorrect class assignments, and PEC-CDC was compared with the baseline CDC under the same noise ratios. The results show that PEC-CDC consistently achieves higher clustering accuracy than CDC across all tested noise levels, indicating that the proposed prediction error-based calibration mechanism can better maintain clustering performance when pseudo-labels are partially corrupted. The specific revisions are shown below:

(**Please refer to Table 7 and the Subsection “Robustness Evaluation under Noisy Pseudo-labels” on Page 19 of the revised manuscript, marked in blue.**)

To further evaluate the robustness of PEC-CDC under challenging self-training conditions, we conducted an additional experiment on CIFAR-10 by injecting random noise into the pseudo-labels during training. This experiment was designed to examine whether the proposed prediction error-based calibration mechanism can maintain reliable clustering performance when pseudo-labels are partially corrupted. Specifically, we randomly replaced a fixed proportion of pseudo-labels with incorrect class assignments and compared PEC-CDC with the baseline CDC under the same noise ratios. All experiments were conducted for 10 epochs using the same random seed. The results are reported in Table 7.

To provide a more complete assessment of practical utility, we added an computational efficiency comparison under the same ResNet-18 backbone. The comparison includes the number of parameters, backbone FLOPs, average training step time, and peak GPU memory consumption. The results show that although PEC-CDC introduces additional parameters due to the PEC module, it maintains favorable computational efficiency. We also added a short analysis explaining that the additional PEC components operate on latent representations, use a frozen teacher, and update only the student associated with the assigned cluster during PEC optimization. The specific revisions are shown below:

As shown in Table 7, PEC-CDC consistently outperforms CDC under all pseudo-label noise ratios. Without injected noise, PEC-CDC achieves an accuracy of 92.73%, compared with 90.25% for CDC. When the noise ratio increases to 40%, PEC-CDC still maintains an accuracy of 91.06%, whereas CDC reaches 89.17%. Across all noise levels, PEC-CDC preserves a performance advantage of approximately 1.56 to 2.61 percentage points over CDC. These results indicate that the proposed prediction error-based calibration mechanism remains effective when pseudo-labels are partially corrupted. Compared with CDC, which relies on calibrated softmax probabilities, PEC-CDC evaluates pseudo-label reliability through sample--cluster distributional consistency. This helps reduce the negative influence of unreliable pseudo-labels during self-training, allowing PEC-CDC to maintain consistently higher clustering performance under noisy pseudo-label conditions.

**(Please refer to Table 6 and the corresponding paragraph in the “Computational Efficiency Comparison” subsection on Page 18-19 of the revised manuscript, marked in blue.)**

To further evaluate the practical utility of PEC-CDC, we compare its training efficiency with representative baseline methods under the same ResNet-18 backbone on CIFAR-10. All methods are evaluated using an input size of 32 $\times$32, a batch size of 256, and an NVIDIA GeForce RTX 2080 Ti GPU. In addition to clustering and classification performance, we report the number of parameters, backbone FLOPs, average training step time, and peak GPU memory consumption.

As shown in Table 6, PEC-CDC contains 21.81M parameters, which is higher than several contrastive baselines and comparable to methods with additional projection or prediction heads, such as SimSiam, VICReg, and Barlow Twins. This increase mainly comes from the additional PEC module, including the frozen teacher and cluster-specific student networks. However, a larger parameter count does not necessarily imply a longer training time. In PEC-CDC, the same ResNet-18 backbone architecture is used across all methods, as reflected by the identical backbone FLOPs. The additional PEC components operate on latent representations rather than full-resolution inputs. Moreover, the teacher network is frozen and does not require gradient updates, and the PEC optimization applies the stop-gradient operation to prevent the regression loss from backpropagating through the encoder. During PEC optimization, each selected sample updates only the student associated with its assigned cluster. Therefore, the extra parameters introduced by the PEC module mainly increase calibration-side storage and introduce only limited additional optimization cost.

PEC-CDC achieves the lowest average training step time of 149.9 ms and the lowest peak GPU memory consumption of 2310.0 MB among the compared methods under the same experimental setting. These results are consistent with the theoretical complexity analysis, which shows that the main additional PEC-related computation for confidence estimation is linear in the number of samples and clusters and does not involve quadratic pairwise comparisons or global graph construction. Therefore, PEC-CDC improves clustering reliability through prediction error-based calibration while maintaining favorable computational efficiency and practical scalability.

To clarify the scalability of PEC-CDC, we added a subsection titled “Computational Complexity Analysis” in the PEC-CDC Framework section. This analysis shows that the main additional computation introduced by the PEC module scales linearly with the number of samples and clusters, and does not involve quadratic pairwise comparisons or global graph construction. The space complexity analysis further explains that the additional memory cost mainly comes from the frozen teacher and cluster-specific student networks, while PEC-CDC avoids quadratic memory growth with respect to the number of samples. The specific revisions are shown below:

(**Please refer to the Computational Complexity Analysis subsection on Pages 15-16 of the revised manuscript, marked in blue.**)

**Computational Complexity Analysis**

We further analyze the computational complexity of PEC-CDC to clarify its scalability relative to the baseline CDC framework. Let $N$ denote the number of unlabeled samples, $C$ denote the number of clusters, and $|\mathcal{S}|$denote the number of selected pseudo-labeled samples in each epoch. We denote the per-sample computational costs associated with the encoder, Clustering Head, frozen teacher, and one cluster-specific student network as $T_{f}$, $T_{clu}$, $T_{t}$, and $T_{s}$, respectively. These terms abstract the corresponding forward or optimization costs required by each stage.

For time complexity, PEC-CDC follows the same self-training backbone as CDC and mainly differs in the confidence estimation stage. In each epoch, weakly augmented samples are first passed through the encoder, requiring $O(NT_{f})$. Then, the PEC module evaluates the prediction error of each sample with respect to all $C$ cluster-specific students. Since the teacher is shared and fixed, this step requires $O(NT_{t} + NCT_{s})$ time, together with $O(NC)$ operations for error-to-confidence conversion and normalization. The dynamic sample selection step sorts samples within clusters according to PEC-derived confidence. Its worst-case cost is$O(N\log N)$, and the practical cost can be reduced by partial top-$K$ selection. After the reliable set $|\mathcal{S}|$ is obtained, clustering optimization requires $O(|\mathcal{S}|(T_{f} + T_{clu}))$, while PEC optimization requires $O(|\mathcal{S}|(T_{f} + T_{t} + T_{s}))$ because each selected sample updates only the student corresponding to its assigned cluster. Therefore, the overall per-epoch time complexity of PEC-CDC can be written as

$O (NT_{f} + NT_{t} + NCT_{s} + NC + N\log N + |\mathcal{S}|(T_{f} + T_{clu} + T_{t} + T_{s})$.

To better justify why prediction error can indicate clustering reliability, we added a new subsection entitled “Theoretical Link Between Prediction Error and Clustering Reliability” in the PEC-CDC Framework section. In this subsection, we provide an assumption-based theoretical analysis to explain the relationship between prediction error and sample--cluster consistency. Specifically, we explain that when a cluster-specific student network is mainly trained on samples from the corresponding cluster distribution, it is expected to approximate the frozen teacher more accurately on the cluster-specific latent support than outside it. Under this assumption, lower prediction error indicates stronger sample--cluster consistency. We also explain that converting prediction error into confidence through a monotonic decreasing function provides a direct link between lower error, higher confidence, and more reliable pseudo-label selection. The specific revisions are shown below:

Compared with CDC, PEC-CDC replaces the probability-based Calibration Head with the PEC module. Thus, the additional time cost mainly comes from evaluating $C$ lightweight student networks during confidence estimation. Importantly, this overhead is linear in both $N$ and $C$, and the method does not require pairwise sample comparisons or global graph construction with $O(N^{2})$ complexity. Since $C$ is fixed by the number of target clusters and the student networks are lightweight heads, the practical overhead remains moderate. This is consistent with the design goal of preserving the scalability of the CDC self-training pipeline while improving the reliability of confidence estimation.

For space complexity, PEC-CDC stores the shared encoder, the Clustering Head, the frozen teacher, and $C$ cluster-specific student networks. Let$P_{f}$, $P_{clu}$, $P_{t}$, and $P_{s}$ denote the numbers of parameters of these components, respectively. The parameter memory of PEC-CDC is therefore

$$O(P_{f} + P_{clu} + P_{t} + CP_{s}).$$

Compared with CDC, whose additional calibration parameters are $P_{cal}$, PEC-CDC replaces the original calibration-side parameter storage with $P_{t} + CP_{s}$. Therefore, the net additional parameter cost is approximately $P_{t} + CP_{s} - P_{cal}$. Since the teacher is frozen and the student networks are implemented as lightweight modules, this additional parameter cost is limited. During dynamic sample selection, PEC-CDC only needs to store tentative labels, confidence scores, and candidate sets, leading to $O(N + CK)$ memory when confidence scores are computed in a streaming or mini-batch manner. In the worst case, storing all sample-cluster confidence scores requires $O(NC)$ memory. Therefore, PEC-CDC avoids quadratic memory growth with respect to $N$ and remains feasible for large-scale deep clustering tasks.

(**Please refer to the newly added “Theoretical Link Between Prediction Error and Clustering Reliability” subsection on Pages 11-12 of the revised manuscript, marked in blue**.)

**Theoretical Link Between Prediction Error and Clustering Reliability**

Since PEC-CDC uses prediction error as the basis of confidence estimation, the key question is why a smaller cluster-specific regression error can indicate stronger sample--cluster consistency and more reliable pseudo-labels. The reliability of prediction error in PEC-CDC can be understood from the teacher--student mechanism adopted in PEC. In PEC, class-specific student networks are trained to approximate a frozen teacher network, and the class with the smallest student--teacher prediction error is selected [20]. When this idea is incorporated into the CDC-style self-training pipeline, prediction error can serve as a confidence-related signal for selecting reliable pseudo-labels, which is consistent with the role of calibrated confidence in CDC [15].

Let $z=f_{\Theta}(x)$denote the latent representation of sample $x$, and let $\mathcal{M}_{c}$ denote the latent support of cluster $c$. In the PEC module, the frozen teacher $h_{\theta}$ provides a fixed target mapping, and each cluster-specific student $g_{\phi^{c}}$is trained using samples assigned to cluster $c$. The prediction error of $x$ with respect to cluster $c$ is defined as

$E_{c}\left( x \right)=\left| \left| g_{\phi^{c}}\left( z \right)-h_{\theta}\left( z \right) \right| \right|^{2}$.

We assume that the samples used to train the $c$-th student are dominated by samples from $\mathcal{M}_{c}$, with bounded contamination from other clusters. Under this condition, the student $g_{\phi^{c}}$ is expected to approximate the teacher more accurately on $\mathcal{M}_{c}$ than on regions far from $\mathcal{M}_{c}$. Formally, for constants $\epsilon_{in} <\epsilon_{out}$, we assume

$\mathbb{E}\left[ E_{c}\left( x \right) \mid z\in\mathcal{M}_{c} \right]\leq\epsilon_{in}$, $\mathbb{E}\left[ E_{c}\left( x \right) \mid z\notin\mathcal{N}_{\rho}\left( \mathcal{M}_{c} \right) \right]\geq\epsilon_{out},$

where $\mathcal{N}_{\rho}\left( \mathcal{M}_{c} \right)$ denotes a $\rho$-neighborhood of $\mathcal{M}_{c}$.

Under this approximation assumption, a lower $E_{c}\left( x \right)$ indicates that sample $x$ is more consistent with the distributional structure learned by the $c$-th student. For a tentative pseudo-label $\hat{y}$, if

$$E_{\hat{y}}\left( x \right)+\Delta< E_{k}\left( x \right), \forall k\neq\hat{y},$$

where $\Delta>0$ is an error margin, then the selected cluster provides stronger distributional support for $x$ than the alternatives. Under this condition, the pseudo-label $\hat{y}$ can be regarded as more reliable. In contrast, noisy samples, samples misassigned at early stages, or samples near ambiguous cluster boundaries are usually not well fitted by a single cluster-specific student. They tend to produce larger errors or smaller error margins, making them less likely to be selected as reliable pseudo-labels.

PEC-CDC converts prediction error into an error-based confidence score through a monotonic decreasing function,

$$conf_{c}\left( x \right)=\exp(-\alpha E_{c}\left( x \right)),$$

where $\alpha>0$. For a fixed cluster $c$, $conf_{c}\left( x \right)$ decreases monotonically as $E_{c}\left( x \right)$ increases, so ranking samples within the same cluster by $conf_{c}\left( x \right)$ is equivalent to ranking them by prediction error in the opposite direction. Moreover, because the subsequent normalization is applied to positive confidence scores, the selected cluster for a given sample is still the one with the smallest prediction error, and a larger error margin generally leads to a higher normalized confidence. This provides a direct link between prediction error and the dynamic selection of reliable pseudo-labels in PEC-CDC.

This analysis also explains the difference between PEC-CDC and probability-based calibration. Softmax confidence reflects relative preference among clusters, while prediction error evaluates whether a sample conforms to the distributional structure learned by a cluster-specific student. This is consistent with the theoretical motivation of PEC, which relates prediction error to uncertainty through Gaussian Process posterior variance [20]. Therefore, within the CDC-style self-training pipeline [15], prediction error serves as an assumption-based reliability signal for sample--cluster consistency and pseudo-label selection.
