# Supplementary material for: PEC-CDC: A prediction error-based calibration framework for robust unsupervised deep clustering
Source: PLoS One. 2026 Jun 25;21(6):e0351959. doi: 10.1371/journal.pone.0351959 (PMC13298931; doi:10.1371/journal.pone.0351959)
Supplement: Peer review history

# Peer review: Decision Letter 0

**DOI:** 10.1371/journal.pone.0351959.r001

**Authors:** Shaofeng Xu

4 May 2026

PONE-D-26-02968

PEC-CDC: A Prediction Error-based Calibration Framework for Robust Unsupervised Deep Clustering

PLOS One

Dear Dr. Li,

Thank you for submitting your manuscript to PLOS ONE. After careful consideration, we feel that it has merit but does not fully meet PLOS ONE’s publication criteria as it currently stands. Therefore, we invite you to submit a revised version of the manuscript that addresses the points raised during the review process.

Please submit your revised manuscript by Jun 18 2026 11:59PM. If you will need more time than this to complete your revisions, please reply to this message or contact the journal office at plosone@plos.org. When you're ready to submit your revision, log on to https://www.editorialmanager.com/pone/ and select the 'Submissions Needing Revision' folder to locate your manuscript file.

Please include the following items when submitting your revised manuscript:

- A letter that responds to each point raised by the academic editor and reviewer(s). You should upload this letter as a separate file labeled 'Response to Reviewers'.
- A marked-up copy of your manuscript that highlights changes made to the original version. You should upload this as a separate file labeled 'Revised Manuscript with Track Changes'.
- An unmarked version of your revised paper without tracked changes. You should upload this as a separate file labeled 'Manuscript'.

If you would like to make changes to your financial disclosure, please include your updated statement in your cover letter. Guidelines for resubmitting your figure files are available below the reviewer comments at the end of this letter.

If applicable, we recommend that you deposit your laboratory protocols in protocols.io to enhance the reproducibility of your results. Protocols.io assigns your protocol its own identifier (DOI) so that it can be cited independently in the future. For instructions see: https://journals.plos.org/plosone/s/submission-guidelines#loc-laboratory-protocols. Additionally, PLOS ONE offers an option for publishing peer-reviewed Lab Protocol articles, which describe protocols hosted on protocols.io. Read more information on sharing protocols at https://plos.org/protocols?utm_medium=editorial-email&utm_source=authorletters&utm_campaign=protocols.

As the corresponding author, your ORCID iD is verified in the submission system and will appear in the published article. PLOS supports the use of ORCID, and we encourage all coauthors to register for an ORCID iD and use it as well. Please encourage your coauthors to verify their ORCID iD within the submission system before final acceptance, as unverified ORCID iDs will not appear in the published article. *Only*   the individual author can complete the verification step; PLOS staff *cannot*   verify ORCID iDs on behalf of authors.

We look forward to receiving your revised manuscript.

Kind regards,

Shaofeng Xu

Academic Editor

PLOS One

Journal Requirements:

When submitting your revision, we need you to address these additional requirements.

1. Please ensure that your manuscript meets PLOS ONE's style requirements, including those for file naming. The PLOS ONE style templates can be found at

https://journals.plos.org/plosone/s/file?id=wjVg/PLOSOne_formatting_sample_main_body.pdf and

https://journals.plos.org/plosone/s/file?id=ba62/PLOSOne_formatting_sample_title_authors_affiliations.pdf.

2. Please note that PLOS One has specific guidelines on code sharing for submissions in which author-generated code underpins the findings in the manuscript. In these cases, we expect all author-generated code to be made available without restrictions upon publication of the work. Please review our guidelines at https://journals.plos.org/plosone/s/materials-and-software-sharing#loc-sharing-code and ensure that your code is shared in a way that follows best practice and facilitates reproducibility and reuse.

3. If the reviewer comments include a recommendation to cite specific previously published works, please review and evaluate these publications to determine whether they are relevant and should be cited. There is no requirement to cite these works unless the editor has indicated otherwise.

Additional Editor Comments (if provided):

(1) Figures 2, 3, and 4 need to be repositioned to appear directly after their first citation in the text. The current placement is misleading and disrupts the reading flow. For instance, Fig. 2 should be moved to Page 16, where the section "Visualization of Representation Evolution" begins.

(2) There is an inconsistency in model naming conventions between the tables and figures. Table 4 uses the identifiers "CD," "PEC-Clu-CDC," and "PEC-CDC," whereas Fig. 2 employs "CDC-Clu" and "PEC." A unified naming scheme should be adopted throughout the manuscript to avoid confusion.

(3) In Fig. 2, the visualization of feature evolution does not reveal sufficiently obvious or interpretable differences between the compared variants. The authors should either provide a more detailed discussion highlighting the specific distinctions, or consider supplementing the figure with quantitative metrics (e.g., silhouette score, Davies–Bouldin index) to support the visual claims.

(4) Table 2 provides a performance comparison across various frameworks in terms of clustering accuracy. However, this represents only one dimension of evaluation. A comparison of training resources and computational efficiency (e.g., training time, GPU memory consumption, FLOPs, or number of parameters) under the same backbone network is also necessary to provide a complete assessment of practical utility.

(5)The method is validated on standard benchmark datasets, which establishes its reliability. However, the evaluation feels incomplete. As the authors themselves state in the manuscript: "PEC-CDC enables more trustworthy and accurate autonomous data discovery in high-dimensional domains such as biomedical and satellite imaging."

I strongly recommend extending the experimental validation to these downstream domains (e.g., biomedical imaging and satellite imaging). Doing so would significantly strengthen the completeness and practical impact of the study.

[Note: HTML markup is below. Please do not edit.]

Reviewers' comments:

Reviewer's Responses to Questions

**Comments to the Author**

1. Is the manuscript technically sound, and do the data support the conclusions?

The manuscript must describe a technically sound piece of scientific research with data that supports the conclusions. Experiments must have been conducted rigorously, with appropriate controls, replication, and sample sizes. The conclusions must be drawn appropriately based on the data presented.

Reviewer #1: Yes

**********

2. Has the statistical analysis been performed appropriately and rigorously?

Reviewer #1: Yes

**********

3. Have the authors made all data underlying the findings in their manuscript fully available?

The PLOS Data policy requires authors to make all data underlying the findings described in their manuscript fully available without restriction, with rare exception (please refer to the Data Availability Statement in the manuscript PDF file). The data should be provided as part of the manuscript or its supporting information, or deposited to a public repository. For example, in addition to summary statistics, the data points behind means, medians and variance measures should be available. If there are restrictions on publicly sharing data—e.g. participant privacy or use of data from a third party—those must be specified.

Reviewer #1: Yes

**********

4. Is the manuscript presented in an intelligible fashion and written in standard English?

PLOS ONE does not copyedit accepted manuscripts, so the language in submitted articles must be clear, correct, and unambiguous. Any typographical or grammatical errors should be corrected at revision, so please note any specific errors here.

Reviewer #1: Yes

**********

5. Review Comments to the Author

Please use the space provided to explain your answers to the questions above. You may also include additional comments for the author, including concerns about dual publication, research ethics, or publication ethics. (Please upload your review as an attachment if it exceeds 20,000 characters)

Reviewer #1: Introduction section

While the motivation for replacing probability metrics with prediction error is compelling, the authors could further strengthen the motivation by explicitly defining the notion of robustness targeted and more clearly articulating the novelty of moving beyond probability-based calibration.

The authors claim robustness in terms of:

-noise tolerance

-avoiding local optima

-stability under ambiguous boundaries

However, the word “robust” still appears more as an outcome than a defined objective. “Robust to what specific perturbations or conditions?”

Related Work Section

While the related work section provides a comprehensive overview of existing self-training and calibration-based deep clustering methods, it would benefit from a more explicit positioning of the proposed approach relative to prior work. In particular, the authors are encouraged to include a summarized comparison (a concise table) that contrasts the proposed PEC-CDC framework with representative prior methods in terms of calibration signal (prediction error vs. probability), reliance on softmax confidence, robustness to noisy pseudo-labels, and computational overhead.

Such a comparison would help clearly establish the novelty and practical advantages of the proposed method over existing approaches

Results and Discussion section

The experimental evaluation is conducted on CIFAR-10, CIFAR-100, and ImageNet-100, which are standard and appropriate benchmarks for unsupervised clustering. However, given the paper’s emphasis on robustness and calibration under noisy pseudo-labels, the current dataset selection primarily reflects clean, in-distribution settings. The authors are encouraged to include additional evaluations under challenging conditions such as noisy or corrupted data, out-of-distribution scenarios to more convincingly demonstrate the robustness and generalizability of the proposed PEC-CDC framework.

The paper does not provide an explicit computational complexity analysis of the proposed PEC-CDC framework. Given that the method introduces an additional PEC Head and modifies the calibration mechanism within a self-training pipeline, it is important to clarify the time and space complexity relative to the baseline CDC and other deep clustering methods. The authors are encouraged to include a theoretical complexity analysis and/or empirical runtime comparisons to demonstrate the scalability and practical feasibility of the proposed approach.

While the paper provides strong empirical motivation and cites prior work suggesting that prediction error correlates with uncertainty, it does not present any theoretical reasoning or formal justification linking prediction error to clustering reliability in the proposed framework. In particular, there is no analysis or assumption explaining why lower prediction error should consistently indicate higher sample–cluster consistency or more reliable pseudo-labels. Even an informal theoretical argument, simplified model, or assumption-based analysis would strengthen the generality and credibility of the proposed calibration mechanism, which currently appears largely empirical.

**********

6. PLOS authors have the option to publish the peer review history of their article (what does this mean?). If published, this will include your full peer review and any attached files.

If you choose “no”, your identity will remain anonymous but your review may still be made public.

**Do you want your identity to be public for this peer review?**   For information about this choice, including consent withdrawal, please see our Privacy Policy.

Reviewer #1: **Yes:**   Patrick Kwabena Mensah

**********

[NOTE: If reviewer comments were submitted as an attachment file, they will be attached to this email and accessible via the submission site. Please log into your account, locate the manuscript record, and check for the action link "View Attachments". If this link does not appear, there are no attachment files.]

To ensure your figures meet our technical requirements, please review our figure guidelines: https://journals.plos.org/plosone/s/figures

You may also use PLOS’s free figure tool, NAAS, to help you prepare publication quality figures: https://journals.plos.org/plosone/s/figures#loc-tools-for-figure-preparation.

NAAS will assess whether your figures meet our technical requirements by comparing each figure against our figure specifications.

# Author comment: Author response to Decision Letter 1

**DOI:** 10.1371/journal.pone.0351959.r002

14 May 2026

Our response to the review comments

First of all, we would like to thank the anonymous reviewers for their constructive comments to improve our manuscript (Manuscript ID: PONE-D-26-02968). We really appreciate their valuable time and great efforts in reviewing the manuscript. Please find below our detailed response to the comments point by point.

Editor：1

Comment 1.1. Figures 2, 3, and 4 need to be repositioned to appear directly after their first citation in the text. The current placement is misleading and disrupts the reading flow. For instance, Fig. 2 should be moved to Page 16, where the section "Visualization of Representation Evolution" begins.

Response to Comment 1.1: We thank the editor for the helpful comment. We have carefully revised the placement of Figs. 2, 3, and 4 to ensure that each figure appears directly after its first citation in the manuscript. Specifically, Fig. 2-4 have also been repositioned after the corresponding paragraphs in which they are first cited. Please refer to Fig. 2 on Page 22, Fig. 3 on Page 23, and Fig. 4 on Page 24 of the revised manuscript.

Comment 1.2. There is an inconsistency in model naming conventions between the tables and figures. Table 4 uses the identifiers "CD," "PEC-Clu-CDC," and "PEC-CDC," whereas Fig. 2 employs "CDC-Clu" and "PEC." A unified naming scheme should be adopted throughout the manuscript to avoid confusion.

Response to Comment 1.2: We thank the editor for pointing out this inconsistency. We have carefully checked the manuscript and unified the naming conventions across the tables, figures, captions, and corresponding textual descriptions. Specifically, the baseline method is now consistently referred to as “CDC,” the ablation variant is referred to as “PEC-Clu-CDC,” and the proposed method is referred to as “PEC-CDC.” In addition, the labels in the visualization figures have been revised to clearly indicate the internal components of PEC-CDC, namely “CluHead of PEC-CDC” and “PEC Module of PEC-CDC,” rather than using the ambiguous terms “CDC-Clu” and “PEC.” These revisions make the terminology consistent and reduce potential confusion for readers. Please refer to Table 9, Figs. 2-4, and the corresponding descriptive paragraphs in the “Visualization of Representation Evolution” section on Pages 21-23 of the revised manuscript, marked in blue, for details.

Comment 1.3. In Fig. 2, the visualization of feature evolution does not reveal sufficiently obvious or interpretable differences between the compared variants. The authors should either provide a more detailed discussion highlighting the specific distinctions, or consider supplementing the figure with quantitative metrics (e.g., silhouette score, Davies–Bouldin index) to support the visual claims.

Response to Comment 1.3: We thank the editor for the constructive suggestion. We have revised the discussion of Fig. 2 to make the interpretation of feature evolution clearer and more quantitative. Specifically, we added two cluster quality metrics, Silhouette Score and Davies-Bouldin Index, to supplement the t-SNE visualization. The added results show that, after training, both the retained Clustering Head and the PEC module achieve higher Silhouette Scores and lower Davies--Bouldin Index values, indicating improved intra-cluster compactness and inter-cluster separability, as shown in Table 9 on Page 21. We also expanded the corresponding analysis to better explain the differences between the initialization and representations after training. These revisions provide quantitative support for the visual claims in Fig. 2. The specific revisions are shown below:

(Please refer to Table 9 and the corresponding paragraph on Page 21 of the revised manuscript, marked in blue, for details.)

Comment 1.4. Table 2 provides a performance comparison across various frameworks in terms of clustering accuracy. However, this represents only one dimension of evaluation. A comparison of training resources and computational efficiency (e.g., training time, GPU memory consumption, FLOPs, or number of parameters) under the same backbone network is also necessary to provide a complete assessment of practical utility.

Response to Comment 1.4: We thank the editor for the helpful suggestion. We agree that clustering accuracy alone is not sufficient to fully evaluate the practical utility of the proposed method. Accordingly, we added a new subsection entitled “Computational Efficiency Comparison” to provide a more comprehensive evaluation under the same ResNet-18 backbone. In this subsection, we compare the number of parameters, backbone FLOPs, average training step time, and peak GPU memory consumption across different methods. The added results show that although PEC-CDC introduces additional parameters due to the PEC module, it maintains favorable computational efficiency, achieving a lower average training step time and lower peak GPU memory consumption under the same experimental setting. We also added a short analysis to explain that the additional PEC components operate on latent representations, use a frozen teacher, and do not involve quadratic pairwise comparisons or global graph construction. The specific revisions are shown below:

(Please refer to Table 6 and the corresponding paragraph in the “Computational Efficiency Comparison” subsection on Page 18-19 of the revised manuscript, marked in blue.)

Comment 1.5. The method is validated on standard benchmark datasets, which establishes its reliability. However, the evaluation feels incomplete. As the authors themselves state in the manuscript: "PEC-CDC enables more trustworthy and accurate autonomous data discovery in high-dimensional domains such as biomedical and satellite imaging." I strongly recommend extending the experimental validation to these downstream domains (e.g., biomedical imaging and satellite imaging). Doing so would significantly strengthen the completeness and practical impact of the study.

Response to Comment 1.5: We thank the reviewer for the constructive suggestion. We agree that evaluation on downstream high-dimensional visual domains can further strengthen the practical impact of the proposed method. Accordingly, we added an additional satellite imaging evaluation on the EuroSAT dataset, which contains Sentinel-2 satellite images from 10 land use and land cover classes. The added experiment evaluates whether PEC-CDC can generalize beyond standard natural image benchmarks to remote sensing scenarios with different visual patterns and spatial structures. The results show that PEC-CDC achieves the best Top-1 accuracy of 94.67% and a Top-5 accuracy of 99.96% under the ResNet-18 backbone, outperforming or matching the compared representative methods. These results indicate that PEC-CDC can learn discriminative representations in satellite imaging scenarios and further demonstrate its practical applicability to downstream high-dimensional data discovery tasks. The specific revisions are shown below:

(Please refer to Table 4 and the fourth and fifth paragraphs of the “Linear Evaluation” section on Page 17 of the revised manuscript, marked in blue.)

Reviewer: 1

Comment 1.1. In Introduction section, while the motivation for replacing probability metrics with prediction error is compelling, the authors could further strengthen the motivation by explicitly defining the notion of robustness targeted and more clearly articulating the novelty of moving beyond probability-based calibration. The authors claim robustness in terms of: noise tolerance, avoiding local optima, stability under ambiguous boundaries. However, the word “robust” still appears more as an outcome than a defined objective. “Robust to what specific perturbations or conditions?”

Response to Comment 1.1: We sincerely thank the reviewer for this insightful comment. We agree that the notion of robustness should be defined more explicitly and that the novelty of moving beyond probability-based calibration should be more clearly articulated. To address this issue, we have revised the manuscript in two sections: the Introduction and the Robustness Evaluation in the Experimental Results and Analysis section.

In the Introduction, we have clarified that the robustness targeted in this study refers to the ability of the model to reduce the selection of unreliable pseudo-labels under noisy or uncertain self-training conditions, including noisy samples, early-stage prediction errors, and samples near ambiguous class boundaries. We also explained that probability-based calibration may still assign high confidence to uncertain samples because softmax confidence is derived from relative class probabilities. The specific revisions are shown below:

(Please refer to the second and third paragraphs in the Introduction on Pages 2-3, marked in blue in the revised manuscript.)

In the Introduction, we further clarified that the novelty of PEC-CDC lies in replacing probability-based confidence with prediction error-based calibration. Instead of evaluating whether a sample obtains a relatively high probability, PEC-CDC evaluates whether the sample conforms to a cluster-specific distributional structure. This revision better explains why prediction error is more suitable for reducing unreliable pseudo-label selection during self-training. The specific revisions are shown below:

(Please refer to the fourth and fifth paragraphs in the Introduction on Page 3, marked in blue in the revised manuscript.)

To further support this motivation, we also added a new subsection entitled “Robustness Evaluation under Noisy Pseudo-labels” in the Experimental Results and Analysis section. In this subsection, we conduct a robustness experiment under noisy pseudo-label settings. Specifically, we injected different proportions of random noise into the pseudo-labels during training on CIFAR-10 and compared PEC-CDC with the baseline CDC under the same noise ratios. The results show that PEC-CDC consistently achieves higher clustering accuracy than CDC across all tested noise ratios. These results demonstrate that PEC-CDC can better maintain clustering performance when pseudo-labels are partially corrupted, confirming its robustness under noisy self-training conditions. The specific revisions are shown below:

(Please refer to Table 7 and the Subsection “Robustness Evaluation under Noisy Pseudo-labels” on Pages 19-20 of the revised manuscript, marked in blue.)

Comment 1.2. In Related Work Section, while the related work section provides a comprehensive overview of existing self-training and calibration-based deep clustering methods, it would benefit from a more explicit positioning of the proposed approach relative to prior work. In particular, the authors are encouraged to include a summarized comparison (a concise table) that contrasts the proposed PEC-CDC framework with representative prior methods in terms of calibration signal (prediction error vs. probability), reliance on softmax confidence, robustness to noisy pseudo-labels, and computational overhead. Such a comparison would help clearly establish the novelty and practical advantages of the proposed method over existing approaches

Response to Comment 1.2: We sincerely thank the reviewer for this constructive suggestion. We agree that a more explicit comparison with representative prior methods is important for clarifying the novelty and practical advantages of PEC-CDC. To address this issue, we have revised the Related Work section by adding a new subsection and a concise comparison table.

In the revised manuscript, we added a new subsection titled “Advantages of PEC-CDC over Representative Prior Methods” to explicitly discuss how PEC-CDC differs from existing methods. Specifically, we compare PEC-CDC with contrastive self-supervised learning methods, non-contrastive self-supervised learning methods, clustering-oriented deep clustering methods, and calibrated deep clustering methods. This added discussion explains that most prior methods either improve representation quality indirectly or rely on prototype assignments, neighborhood consistency, or probability-based confidence for pseudo-label selection. In contrast, PEC-CDC uses prediction error to evaluate sample--cluster distributional consistency, which helps reduce the selection of unreliable pseudo-labels during self-training. The specific revisions are shown below:

(Please refer to the newly added subsection in the Related Work section on Page 5 of the revised manuscript, marked in blue.)

We also added a concise comparison table to present the differences between PEC-CDC and representative prior methods more clearly. The table compares the methods in terms of calibration signal, reliance on softmax confidence, robustness to noisy pseudo-labels, and computational overhead, as suggested by the reviewer. This comparison shows that PEC-CDC differs from prior methods by using prediction error rather than softmax-derived probability as the calibration signal. It also illustrates that PEC-CDC reduces reliance on softmax confidence, improves robustness to noisy pseudo-labels, and maintains moderate computational overhead. The specific revisions are shown below:

(Please refer to Table 1 in the Related Work section on Page 6 of the revised manuscript, marked in blue.)

Comment 1.3. In Results and Discussion section, the experimental evaluation is conducted on CIFAR-10, CIFAR-100, and ImageNet-100, which are standard and appropriate benchmarks for unsupervised clustering. However, given the paper’s emphasis on robustness and calibration under noisy pseudo-labels, the current dataset selection primarily reflects clean, in-distribution settings. The authors are encouraged to include additional evaluations under challenging conditions such as noisy or corrupted data, out-of-distribution scenarios to more convincingly demonstrate the robustness and generalizability of the proposed PEC-CDC framework. The paper does not provide an explicit computational complexity analysis of the proposed PEC-CDC framework. Given that the method introduces an additional PEC Head and modifies the calibration mechanism within a self-training pipeline, it is important to clarify the time and space complexity relative to the baseline CDC and other deep clustering methods. The authors are encouraged to include a theoretical complexity analysis and/or empirical runtime comparisons to demonstrate the scalability and practical feasibility of the proposed approach. While the paper provides strong empirical motivation and cites prior work suggesting that prediction error correlates with uncertainty, it does not present any theoretical reasoning or formal justification linking prediction error to clustering reliability in the proposed framework. In particular, there is no analysis or assumption explaining why lower prediction error should consistently indicate higher sample–cluster consistency or more reliable pseudo-labels. Even an informal theoretical argument, simplified model, or assumption-based analysis would strengthen the generality and credibility of the proposed calibration mechanism, which currently appears largely empirical.

Response to Comment 1.3: We sincerely thank the reviewer for these insightful and constructive comments. We agree that the robustness, scalability, and theoretical basis of PEC-CDC should be further clarified to strengthen the credibility of the proposed framework. To address these concerns, we have revised the manuscript in four specific parts: Robustness Evaluation, Computational Efficiency Comparison, Computational Complexity Analysis, and the Theoretical Link Between Prediction Error and Clustering Reliability.

To evaluate PEC-CDC under more challenging self-training conditions, we added an experiment on CIFAR-10 with noisy pseudo-labels. Specifically, different proportions of pseudo-labels were randomly replaced with incorrect class assignments, and PEC-CDC was compared with the baseline CDC under the same noise ratios. The results show that PEC-CDC consistently achieves higher clustering accuracy than CDC across all tested noise levels, indicating that the proposed prediction error-based calibration mechanism can better maintain clustering performance when pseudo-labels are partially corrupted. The specific revisions are shown below:

(Please re

Attachment Submitted filename: Response to Reviewers.docx

# Editor report: Decision Letter 1

**DOI:** 10.1371/journal.pone.0351959.r003

**Authors:** Shaofeng Xu

3 Jun 2026

PEC-CDC: A Prediction Error-based Calibration Framework for Robust Unsupervised Deep Clustering

PONE-D-26-02968R1

Dear Dr. Li,

We’re pleased to inform you that your manuscript has been judged scientifically suitable for publication and will be formally accepted for publication once it meets all outstanding technical requirements.

Within one week, you’ll receive an e-mail detailing the required amendments. When these have been addressed, you’ll receive a formal acceptance letter and your manuscript will be scheduled for publication.

An invoice will be generated when your article is formally accepted. Please note, if your institution has a publishing partnership with PLOS and your article meets the relevant criteria, all or part of your publication costs will be covered. Please make sure your user information is up-to-date by logging into Editorial Manager at Editorial Manager® and clicking the ‘Update My Information' link at the top of the page. For questions related to billing, please contact billing support.

If your institution or institutions have a press office, please notify them about your upcoming paper to help maximize its impact. If they’ll be preparing press materials, please inform our press team as soon as possible -- no later than 48 hours after receiving the formal acceptance. Your manuscript will remain under strict press embargo until 2 pm Eastern Time on the date of publication. For more information, please contact onepress@plos.org.

Kind regards,

Shaofeng Xu

Academic Editor

PLOS One

Additional Editor Comments (optional):

Reviewers' comments:

# Editor report: Acceptance letter

**DOI:** 10.1371/journal.pone.0351959.r004

**Authors:** Shaofeng Xu

PONE-D-26-02968R1

PLOS One

Dear Dr. Li,

I'm pleased to inform you that your manuscript has been deemed suitable for publication in PLOS One. Congratulations! Your manuscript is now being handed over to our production team.

At this stage, our production department will prepare your paper for publication. This includes ensuring the following:

* All references, tables, and figures are properly cited

* All relevant supporting information is included in the manuscript submission,

* There are no issues that prevent the paper from being properly typeset

You will receive further instructions from the production team, including instructions on how to review your proof when it is ready. Please keep in mind that we are working through a large volume of accepted articles, so please give us a few days to review your paper and let you know the next and final steps.

Lastly, if your institution or institutions have a press office, please let them know about your upcoming paper now to help maximize its impact. If they'll be preparing press materials, please inform our press team within the next 48 hours. Your manuscript will remain under strict press embargo until 2 pm Eastern Time on the date of publication. For more information, please contact onepress@plos.org.

You will receive an invoice from PLOS for your publication fee after your manuscript has reached the completed accept phase. If you receive an email requesting payment before acceptance or for any other service, this may be a phishing scheme. Learn how to identify phishing emails and protect your accounts at https://explore.plos.org/phishing.

If we can help with anything else, please email us at customercare@plos.org.

Thank you for submitting your work to PLOS ONE and supporting open access.

Kind regards,

PLOS ONE Editorial Office Staff

on behalf of

Dr. Shaofeng Xu

Academic Editor

PLOS One
